# Genome insight and probiotic potential of three novel species of the genus *Corynebacterium*

**DOI:** 10.3389/fmicb.2023.1225282

**Published:** 2023-07-06

**Authors:** Md Shamsuzzaman, Ram Hari Dahal, Shukho Kim, Jungmin Kim

**Affiliations:** ^1^Department of Biomedical Science, School of Medicine, Kyungpook National University, Daegu, Republic of Korea; ^2^Department of Microbiology, School of Medicine, Kyungpook National University, Daegu, Republic of Korea

**Keywords:** *Corynebacterium intestinale* sp. nov., *Corynebacterium stercoris* sp. nov., *Corynebacterium faecium* sp. nov., probiotic, antimicrobial agent, antioxidant activities

## Abstract

Three bacterial strains, B5-R-101^T^, TA-R-1^T^, and BL-R-1^T^, were isolated from the feces of a healthy Korean individual. Cells of these strains were Gram-stain-positive, facultatively anaerobic, oxidase-negative, catalase-positive, rod-shaped, and non-motile. They were able to grow within a temperature range of 10–42°C (optimum, 32–37°C), at a pH range of 2.0–10.0 (optimum, pH 5.5–8.0), and at NaCl concentration of 0.5–10.5% (w/v). All the three strains exhibited 2,2-Diphenyl-1-picrylhydrazyl (DPPH) radical scavenging activities ranging from 58 ± 1.62 to 79 ± 1.46% (% inhibition). These strains survived in lower pH (2.0) and in 0.3% bile salt concentration for 4 h. They did not show hemolytic activity and exhibited antimicrobial activity against pathogenic bacteria, such as *Escherichia coli*, *Acinetobacter baumannii*, *Staphylococcus aureus*, and *Salmonella enterica*. The genomic analysis presented no significant concerns regarding antibiotic resistance or virulence gene content, indicating these strains could be potential probiotic candidates. Phylogenetic analysis showed that they belonged to the genus *Corynebacterium*, with 98.5–99.0% 16S rRNA gene sequence similarities to other members of the genus. Their major polar lipids were diphosphatidylglycerol and phosphatidylglycerol. The abundant cellular fatty acids were C_16:0_, C_18:1_*ω*9*c*, and anteiso-C_19:0_. Genomic analysis of these isolates revealed the presence of genes necessary for their survival and growth in the gut environment, such as multi-subunit ATPases, stress response genes, extracellular polymeric substance biosynthesis genes, and antibacterial genes. Furthermore, the genome of each strain possessed biosynthetic gene clusters with antioxidant and antimicrobial potentials, including terpenes, saccharides, polyketides, post-translationally modified peptides (RIPPs), and non-ribosomal peptides (NRPs). *In silico* DNA–DNA hybridization (dDDH) and average nucleotide identity (ANI) values were lower than the thresholds to distinguish novel species. Based on phenotypic, genomic, phylogenomic, and phylogenetic analysis, these potential probiotic strains represent novel species within the genus *Corynebacterium*, for which the names *Corynebacterium intestinale* sp. nov. (type strain B5-R-101^T^ = CGMCC 1.19408^T^ = KCTC 49761^T^), *Corynebacterium stercoris* sp. nov. (type strain TA-R-1^T^ = CGMCC 1.60014^T^ = KCTC 49742^T^), and *Corynebacterium faecium* sp. nov. (type strain BL-R-1^T^ = KCTC 49735^T^ = TBRC 17331^T^) are proposed.

## Highlights

– We have isolated, identified, and characterized three novel species of the genus *Corynebacterium* from faecal samples of a healthy human.
– These isolates exhibited antioxidant and antimicrobial activities and have potential probiotic properties, making them a promising prospect for the development of new therapies for chronic diseases and human gut microbiota dysbiosis.
– These novel isolates of the genus *Corynebacterium*, which exhibit probiotic properties, could be a basis for future research into human gut bacteria and human health.
– Additionally, the discovery of previously uncultured species of the genus *Corynebacterium* may help in resolving the taxonomy of the human gut microbiome.

## 1. Introduction

The members of the genus *Corynebacterium* are gram-positive, rod-shaped, non-sporulating microbes that contain unique mycolic acids in their cell walls. They are widely ubiquitous in the nature and can be isolated from soil, water, plants, humans, and animals (von Graevenitz and Bernard, 2006). Currently, there are 155 species of the genus *Corynebacterium* with a validly published and correct name (https://lpsn.dsmz.de/genus/corynebacterium, accessed 27 June 2023). Recently, members of the genus *Corynebacterium* have gained attention for their remarkable health benefits, with species such as *C*. *parvum*, *C*. *accolens*, and *C*. *simulans* being identified as potential probiotics for improving gut health (Alak et al., 1997; Huang et al., 2022). *Corynebacterium glutamicum* produces antimicrobial peptides that lower cholesterol levels and inhibit pathogenic bacteria in their human hosts (Kataoka et al., 2019). *Corynebacterium pseudotuberculosis* stimulates cytokine production to activate the immune system (De Souza et al., 2014). Additionally, *C*. *accolens* produces enzymes that inhibit the growth of *Cutibacterium acne*, which causes acne (Bomar et al., 2016). *Corynebacterium urealyticum* reduces the risk of urinary tract infections (UTIs; Chapartegui-González et al., 2020) and *C*. *kroppenstedtii* improves atopic dermatitis symptoms (Wong et al., 2017). In contrast, some species of the genus *Corynebacterium* are opportunistic pathogens that cause human diseases. For example, *C*. *diphtheriae* is responsible for the eradicated disease diphtheria, which has been eliminated in developed countries through vaccination programs targeting the primary virulence factor, diphtheria toxin (Sutcliffe et al., 2018). Other species of the genus *Corynebacterium*, such as *C*. *ulcerans*, *C*. *pseudodiphtheriticum*, *C*. *striatum*, *C*. *riegelii*, *C*. *glucuronolyticum*, *C*. *urealyticum*, and *C*. *jeikeium*, can cause various infections, including respiratory infections, UTIs, skin infections, endocarditis, and septic arthritis (Sato and Matsui, 2011; Cazanave et al., 2012).

Free radicals can damage vital macromolecules such as nucleic acids, lipids, carbohydrates, and proteins, leading to serious health problems such as cancer, atherosclerosis, and premature aging (Phaniendra et al., 2015). Antioxidants play a crucial role in human health by protecting cells from damage caused by free radicals and oxidative stress. Incorporating antioxidants into one’s diet or taking supplements can help lower the risk of various diseases (Martemucci et al., 2022). The search for safe and natural sources of antioxidants has led to studies on plants, vegetables, spices, and fruits (Rahaman et al., 2023) aimed at discovering those with antioxidant compounds. Microorganisms can provide a high fluence of natural bioactive molecules for food and nutraceutical applications as they can be cultivated under controlled conditions at a much faster rate than plants (Chandra et al., 2020). Microbial antioxidant systems play a significant role in maintaining the cellular redox balance and protecting against oxidative stress caused by reactive oxygen species (ROS) and reactive nitrogen species (RNS; Dahal et al., 2020). The defense mechanisms include enzymatic and nonenzymatic antioxidant systems such as those involving superoxide dismutase, catalase, glutathione, and thioredoxin. Together, these systems neutralize ROS and RNS and maintain the redox balance within the cell. Furthermore, bacterial antioxidant systems play a crucial role in numerous cellular processes, such as gene regulation, signal transduction, and virulence (Kim et al., 2022). Importantly, the novel member of the genus *Corynebacterium* having antioxidant properties may have significant role to combat with oxidative stress and maintain the gut homeostasis (Rajoka et al., 2021).

Microbes contain numerous bioactive metabolites, including new antibiotics that exhibit potent antimicrobial properties against multidrug-resistant bacteria. Because of the emergence of drug-resistant pathogens in humans, antimicrobial resistance has become a major public health concern. This underscores an urgent need to develop new antimicrobial agents that can improve the outcomes of infectious diseases and ultimately save lives (Serwecińska, 2020). Although bioactive compounds derived from plants have been used for many years to treat human infections, they are becoming less effective in combating some ailments (Lamponi et al., 2023). However, microorganisms still provide a rich source of untapped biomolecules with diverse structural and functional antimicrobial activities. Modern techniques, such as advanced molecular biology techniques, omics technology, machine learning, and cost-effective and convenient model organisms, have made it more feasible to search for new antimicrobial drugs and identify novel drug targets. This approach is promising for discovering novel therapies to combat infectious diseases (Serwecińska, 2020; Danquah et al., 2022; Sundararaman and Halami, 2022). Recent studies have shown that *C*. *accolens* has antimicrobial activity against *Streptococcus pneumoniae*, *C*. *pseudodiphtheriticum* is effective against both *Staphylococcus aureus* and *Moraxella catarrhalis*, and *C*. *propinquum* can fight coagulase-negative staphylococci. These findings suggest that multiple species of *Corynebacterium* may be effective against infections of the upper respiratory tract as they target different pathogens through distinct mechanisms (Lappan and Peacock, 2019).

The World Health Organization describes probiotics as viable microorganisms that are nonpathogenic and can be administered in specific quantities to enhance health (Binda et al., 2020). Probiotic microorganisms can play a role in managing several health conditions, including irritable bowel disease, hypertension, constipation, irritable bowel syndrome, diarrhea, allergies, and diabetes (Alkalbani et al., 2019; Wang et al., 2019). Probiotic strains should have desirable characteristics, including the ability to survive in stomach and intestinal acids, ability to attach to cells in the gut (Sundararaman et al., 2021), and having no harmful genes or activity. Additionally, they should exhibit antimicrobial activity and withstand the fermentation and storage process (Binda et al., 2020). Although not all the characteristics mentioned above are necessary for potential probiotics, having qualities such as heat tolerance and the ability to produce exopolysaccharides (EPS) is desirable (Elmansy et al., 2022). Probiotic strains can make functional foods with superior health benefits. Because of their health benefits, research to find new probiotics from different sources is ongoing (Nagpal et al., 2012). Most importantly, probiotics for human use should originate from humans (Dahal et al., 2023).

This study aimed to isolate and characterize, from feces of a healthy human, novel bacterial species that have antioxidant, antimicrobial, and probiotic properties for treating and preventing various human ailments. We isolated and evaluated the probiotic potential of novel strains B5-R-101^T^, TA-R-1^T^, and BL-R-1^T^, as well as their antibacterial and nonhemolytic activities and ability to tolerate harsh conditions, such as low pH and high salt concentrations. Additionally, we conducted a comprehensive analysis using whole-genome sequencing and comparative genomic analysis to explore the genetic characteristics of probiotic strains. Furthermore, we investigated their antioxidant properties to determine their ability to combat oxidative stress. We identified novel species by analyzing the 16S rRNA gene sequences. A total of 97 strains from 10 healthy Korean individuals have been isolated aerobically. Among them three potential novel strains B5-R-101^T^, TA-R-1^T^, and BL-R-1^T^ belonging to the genus *Corynebacterium* have been evaluated for the probiotic potentials and its taxonomic status. A neighbor-joining tree was reconstructed to compare validly published species of the genus *Corynebacterium*. We also determined the dDDH and ANI values to identify and validate the status of the novel species. Finally, through phenotypic, genomic, and phylogenetic analyses, we proposed three novel species of the genus *Corynebacterium* exhibiting probiotic, antimicrobial, and antioxidant properties. Our findings have significant implications for the development of new probiotic and antioxidant therapies that can enhance human health.

## 2. Materials and methods

### 2.1. Isolation, ecology, and preservation

Fresh fecal sample collected from a healthy Korean individual at Kyungpook National University Hospital (KNUH) was placed in an anaerobic gas pouch (BD GasPak EZTM Pouch Systems, BD, NJ, United States) and transported to an anaerobic gas chamber in the laboratory. To enrich the fecal samples, 1 g of feces was mixed with 9 mL of defibrinated sheep blood and incubated under aerobic and anaerobic conditions at 37°C for 2 days. The remaining samples were stored at −70°C for further analysis. Serial dilutions were made up to 10^9^ in phosphate-buffered saline, and 100 μL of each enriched fecal sample (10^5^–10^9^) was plated on brain heart infusion agar (BHA) with 5% of defibrinated sheep blood and incubated at 37°C for 3–5 days. Pure colonies were obtained by selecting and streaking each colony on a new blood agar plate until a pure colony was obtained. The purified colonies were stored at −70°C in a 50% glycerol stock for long-term preservation and have been deposited in different culture collection centers such as Korean Collection for Type Cultures (KCTC), China General Microbiological Culture Collection (CGMCC), and Thailand Bioresource Research Center (TBRC).

### 2.2. Phylogenetic analysis

Genomic DNA was extracted using the method described previously (Dahal et al., 2021). The 16S rRNA gene fragment was amplified using the universal primers 27F and 1492R (Frank et al., 2008). Sequencing was performed with an Applied Biosystems 3770XL DNA analyzer and the Big Dye Terminator cycle sequencing kit v.3.1 (Applied Biosystems). We compared the obtained sequences to the available 16S rRNA gene sequences on the EzBioCloud server (Yoon et al., 2017; https://www.ezbiocloud.net). Phylogenetic trees were reconstructed using the software package MEGA 11 (https://www.megasoftware.net/; Tamura et al., 2021), and multiple alignments of the all sequences were performed using the SINA^1^ prior to phylogenetic tree reconstruction with three tree making algorithms: neighbor-joining (Saitou and Nei, 1987), maximum-likelihood (Felsenstein, 1981), and maximum-parsimony (Fitch, 1971). The evolutionary distances were calculated according to Kimura 2-parameter model (Kimura, 1980) and bootstrap analysis was based on 1,000 replications (Felsenstein, 1985).

### 2.3. Genome analysis

For whole-genome sequencing, genomic DNA was extracted utilizing DNeasy Blood and Tissue kits (Qiagen; Hilden, Germany). Whole-genome shotgun sequencing of strains B5-R-101^T^, TA-R-1^T^, and BL-R-1^T^ was conducted by Macrogen (Seoul, Republic of Korea) utilizing the Illumina HiSeq platform. The resulting sequences were assembled using SPAdes (Bankevich et al., 2012). The accuracy of the genome assembly was verified by comparing the 16S rRNA gene sequences through the NCBI Align Sequences Nucleotide BLAST tool (Zhang et al., 2004). The potential contamination of each genome sequences was assessed using the ContEst16S server.^2^ Using subsystem technology, we annotated the genome data of the strains B5-R-101^T^, TA-R-1^T^, and BL-R-1^T^ on the Rapid Annotation server^3^ and the NCBI Prokaryotic Genome Annotation Pipeline.^4^ We performed multiple genome alignments in the presence of large-scale evolutionary events using MAUVE^5^ and used the CGView server to create genomic circular feature maps.^6^ We also annotated the functions of predicted coding genes using the database for carbohydrate-active enzymes (CAZymes) and the Kyoto Encyclopedia of Genes and Genomes. To identify antibiotic resistance genes, we used the comprehensive antibiotic resistance database^7^ and the antiSMASH database to predict the presence of gene clusters encoding secondary metabolites.^8^ We used the OrthoANI tool^9^ to compute the average nucleotide identity (ANI) among genomes from closest species of the genus *Corynebacterium*. Additionally, Genomic-to-Genomic Distance Calculator (GGDC 3.0; http://ggdc.dsmz.de/ggdc.php) was used to determine the digital DNA–DNA Hybridization (dDDH) values following the recommended formula-2.0 (Meier-Kolthoff et al., 2022). The phylogenomic tree was constructed using the genome server.^10^ Additionally, the phylogenomic tree was reconstructed using the up-to-date bacterial core gene set (UBCG v.3; http://www.ezbiocloud.net/tools/ubcg; Na et al., 2018). The G + C content of DNA was calculated using the whole-genome sequences.

### 2.4. Biochemical and morphological characterization

The morphology and size of each cell were visualized using a Transmission Electron Microscope (TEM, JEM-1011). Gram staining was performed using a reagent kit (Sigma, St. Louis, MO, United States). Physiological tests were conducted to assess the growth potential of the strains at different temperatures (4, 10, 15, 20, 25, 30, 37, 40, 42, and 50°C). Bacterial growth was determined on brain heart infusion agar (BHA; BD), BHA with 5% defibrinated sheep blood, Luria-Bertani agar (LBA; BioShop), and Muller-Hinton agar (MHA; BD). Salt tolerance was observed in BHI broth supplemented with NaCl concentrations ranging from 1 to 11% (w/v). Catalase test was assessed using 3% (v/v) hydrogen peroxide and oxidase activity was determined using 1% (w/v) tetra-methyl-*p*-phenylenediamine dihydrochloride. The hydrolysis of gelatin, starch, casein, tyrosine, and Tweens 20, 40, and 80 were performed as described previously (Dahal et al., 2021). Other biochemical and physiological tests were determined using API ZYM, API 20NE, and API 50CH test kits (bioMérieux) according to the manufacturer’s instructions.

### 2.5. Survival under low pH

Growth in low pH (acid tolerance) was tested according to the method described by Shokryazdan et al. (2014) with fewer modifications. Cells of each strain (B5-R-101^T^, TA-R-1^T^, and BL-R-1^T^; in a final concentration of 8 log CFU/mL) were inoculated aerobically into BHI broth with pH ranging from 2.0 to 6.0 (adjusted with 1 N HCl). After incubating the cultures at 37°C for 48 h, their survival rate was evaluated by measuring the bacterial density at 5-h intervals using OD absorbance at 600 nm. Each experiment was triplicated and was performed twice.

### 2.6. Bile salt tolerance

For analysis of bile salt tolerance, we followed the procedure described previously by Nami et al. (2019) with a few minor modifications. All the three strains B5-R-101^T^, TA-R-1^T^, and BL-R-1^T^ were inoculated in BHI broth containing bile salt (Sigma-Aldrich; St. Louis, Missouri, United States) at different concentrations [0.3 and 0.5% (w/v)] and incubated at 37°C for 0–4 h. Colony forming unit (CFU) was counted for every 0, 1, 3, and 4 h to calculate the survival rate. The survival rate in the bile environment was estimated by comparing the number of viable CFU using the following equation:


$$\mathrm{Survival rate}\;\left(\%\right)=\left(N/N_{0}\right)\times 100$$

where *N* = number of viable CFU at different time interval, *N*_0_ = initial number of CFU.

Each experiment was triplicated.

### 2.7. Antimicrobial activity

The antibacterial capabilities of cell-free supernatant (CFS) were examined using the method by Lim et al. (2018) with modifications. Strains B5-R-101^T^, TA-R-1^T^, and BL-R-1^T^ were well grown in the BHI broth for 24 h at 37°C. To prepare the CFS, the bacterial cultures were first centrifuged at 10,000 × *g* for 15 min at 4°C before the supernatant was collected and filtered through a 0.22-micron filter (Millipore Nihon, United States). The resulting CFS was tested for its effect on the growth of pathogenic microorganisms (*Escherichia coli* ATCC 25922, *E*. *coli* KBN 7288, *E*. *coli* KBN 4004, *Salmonella enterica* PT4, *Acinetobacter baumanii* ATCC 17978, and *Staphylococcus aureus* ATCC 25923) in broth. CFS assay was assessed in 96-well microplates (SPL Life Sciences Co., Ltd.; Pocheon; Republic of Korea) as described by Wang et al. (2017) with some modifications. A 100-μL suspension containing an estimated 2 × 10^5^ CFU of pathogenic bacteria in BHI broth was mixed with 100 μL of CFS in each sterile microplate. The mixture was incubated under aerobic conditions for 24 h at 37°C. Optical density at 600 nm was measured by VersaMax microplate reader (Molecular Devices, LLC, San Jose, CA, United States). For the negative control, only BHI broth was used as for the positive control BHI broth with tetracycline (10 μg/mL) was used.

### 2.8. Antibiotic susceptibility testing

Antibiotic susceptibility testing is a laboratory method used to determine the susceptibility of a bacterial strain to a specific antibiotic or group of antibiotics. The disk diffusion method was used to perform antibiotic susceptibility testing. Fresh bacterial cultures were spread on BHA agar plates before antibiotic disks were placed on the agar plates. The plates were subsequently incubated for 24 h at 37°C. *E*. *coli* ATCC 25922 was used as a control. The antibiotic susceptibility testing was performed with the following antibiotics: ampicillin (10 μg), gentamicin (10 μg), ceftazidime (30 μg), erythromycin (15 μg), piperacillin (10 μg), cefoxitin (30 μg), colistin (10 μg), cefotaxime (30 μg), amikacin (30 μg), aztreonam (30 μg), ciprofloxacin (5 μg), and oxacillin (15 μg).

### 2.9. Hemolytic activity

The hemolytic activity of the strains was evaluated on BHA with 5% defibrinated sheep blood for 48 h at 37°C. Strains exhibiting greenish zones around the colonies (*α*-hemolysis) or no effect on the blood plates (*γ*-hemolysis) were interpreted as nonhemolytic. Strains that displayed blood lysis zones around the colonies were classified as hemolytic (*β*-hemolysis; Pieniz et al., 2014).

### 2.10. Chemotaxonomic characteristics

Respiratory quinones and polar lipids were extracted from freeze-dried cells using the method described by Minnikin et al. (1984). The two-dimensional thin-layer chromatography method was used to extract and identify polar lipids. Cellular fatty acids of three isolates along with reference strains under the identical conditions was extracted and analyzed using the Sherlock Microbial Identification System (version 6.0.1, MIDI Inc., Newark, DE, United States; Sasser, 1990).

### 2.11. Scavenging activity on 2,2-diphenyl-1-picrylhydrazyl radicals

To prepare the inhibitor, well-grown bacteria in BHI medium at 4°C were centrifuged. A reaction mixture of 180 μL consisting of 90 μL of 0.1 mM 2,2-diphenyl-1-picrylhydrazyl (DPPH) dissolved in methanol (MeOH) and 90 μL of the sample solution was prepared. Supplementary Table S1 shows how the test reaction mixture was prepared. The reaction was incubated at 37°C for 30 min in 96-well plates (Dahal et al., 2020). Ascorbic acid (vitamin C) was used as a positive control. The test reaction was mixed thoroughly and incubated in the dark for 30 min at 37°C in 96-well microplate. The absorption at 516 nm was measured using a VersaMax microplate reader (Molecular Devices, LLC, San Jose, CA, United States). The proportion of radicals tripled after each treatment. The following formula was used to calculate scavenging activities: Bacterial absorbance − media absorbance = original absorbance.

In this study, DPPH radical scavenging activity was calculated by the following formula:


[DPPH inhibition ()]=[(Ac−As)/Ac]×100

where Ac and As are the absorbances of the control and sample, respectively.

## 3. Results

### 3.1. Biochemical and morphological characterization

Strains B5-R-101^T^, TA-R-1^T^, and BL-R-1^T^ were Gram-stain-positive facultative anaerobes that were non-motile, catalase-positive, and oxidase-negative. These strains grew well on BHA with 5% defibrinated sheep blood (blood agar); weakly on MHA and LBA. The colonies on blood agar were orange in color and displaying a smooth, convex, and opaque morphology. Cells were rod shaped (Supplementary Figure S1). They were able to grow within a temperature range of 10–42°C (except strain TA-R-1^T^ which poorly grew at 45°C), at pH levels ranging from 2.0 to 10.0, and at NaCl concentrations ranging from 0 to 10.5% (w/v). The distinct physiological and biochemical characteristics that differentiated strains B5-R-101^T^, TA-R-1^T^, and BL-R-1^T^ from their closest phylogenetic neighbors are given in Table 1.

**Table 1:** Phenotypic characteristics of the proposed novel strains of the genus *Corynebacterium* are distinct from those of phylogenetically related type strains.

Characteristic	1	2	3	4	5	6	7	8	9	10
Highest salt tolerance (%, w/v)	10.5	9.5	10.5	8	10	10	9	10	10	10.5
pH range	2.0–10.0	2.0–10.0	2.0–9.0	3.0–8.0	3.5–10.0	3.5–10.0	3.0–10.0	2.5–10.0	3.0–10.0	3.0–8.0
Hydrolysis:										
Starch	−	−	+	−	−	−	−	+	+	−
Urea	−	−	+	−	−	−	−	−	−	−
Gelatin	+	+	+	+	−	−	+	+	+	+
Enzyme activities (API ZYM):										
Lipase (C14)	+	+	+	−	+	+	+	+	+	−
Leucine arylamidase	+	+	+	−	+	+	+	+	+	−
Valine arylamidase	+	+	+	−	+	+	+	+	+	−
Cystine arylamidase	+	+	+	−	+	+	+	+	+	−
Trypsin	+	+	+	−	+	+	+	−	−	−
*α*-chymotrypsin	+	+	+	+	+	−	−	−	−	−
Acid phosphatase	+	+	+	+	+	+	+	−	−	+
*ß*-glucosidase	−	+	−	−	−	−	−	−	−	−
Acid production from:										
l-arabinose	−	−	w	w	−	−	−	−	−	w
d-ribose	−	−	w	w	−	−	−	−	−	w
d-xylose	−	−	−	w	−	−	−	−	−	w
d-adonitol	−	−	w	w	−	−	−	−	−	w
d-glucose	w	+	w	w	+	w	w	w	w	w
d-fructose	w	w	+	−	+	−	−	−	−	−
d-mannose	w	+	−	−	−	−	−	−	w	−
l-rhamnose	−	−	+	w	w	−	−	−	−	w
Arbutin	w	w	−	w	w	−	−	−	−	w
d-maltose	w	+	+	−	−	−	−	−	−	−
d-saccharose (sucrose)	w	w	+	−	−	−	−	−	−	−
Gluconate	−	−	+	w	−	−	−	−	−	w
2-ketogluconate	w	w	+	−	w	w	w	w	w	−

Strains: 1, B5-R-101^T^; 2, TA-R-1^T^; 3, BL-R-1^T^; 4, *C*. *aurimucosum* DSM 44532^T^; 5, *C*. *minutissimum* DSM 20651^T^; 6, *C*. *singulare* DSM 44357^T^; 7, *C*. *aquatimens* DSM 45632^T^; 8, *C*. *tuscaniense* DSM 45101^T^; 9, *C*. *ureicelerivorans* DSM 45051^T^; and 10, *C*. *mucifaciens* DSM 44265^T^. All the data were obtained from this study. +, positive; w, weakly positive; −, negative.

### 3.2. 16S rRNA phylogeny

**Figure 1:**
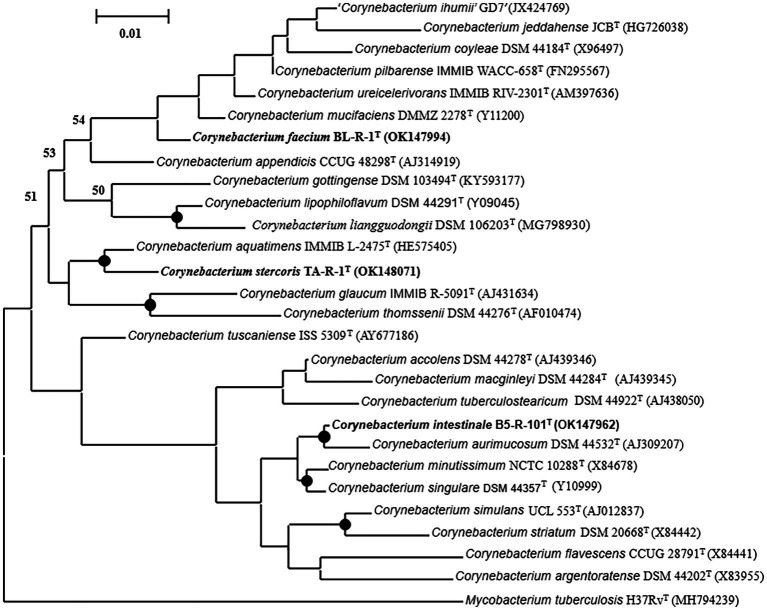
A maximum-likelihood tree reconstructed using the almost complete 16S rRNA gene sequences to display the phylogenetic position of the strains B5-R-101^T^, TA-R-1^T^, and BL-R-1^T^ to their closely related members of the genus *Corynebacterium*. Nodes consistently recovered by all three treeing methods (neighbor-joining, maximum-likelihood, and maximum-parsimony) are represented by filled circles. The percentage of 1,000 bootstrap replicates are indicated by node numbers, with only values greater than or equal to 50% displayed. The out-group used was *Mycobacterium tuberculosis* H37Rv^T^. Nucleotide accession numbers are given in parentheses. The bar represents 0.01 substitutions per nucleotide position.

The 16S rRNA gene sequence was analyzed to determine the phylogenetic relationship of the novel strains with closest species of the genus *Corynebacterium*. Based on the analysis, strain B5-R-101^T^ TA-R-1^T^, and BL-R-1^T^ showed the highest 16S rRNA gene sequence similarities to the following strains: *C*. *aquatimens* IMMIB L-2475^T^, *C*. *aurimucosum* NRRLB-24143^T^, *C*. *minutissimum* ATCC 23348^T^, *C*. *mucifaciens* DMMZ 2278^T^, *C*. *singulare* IBS B-52218^T^, *C*. *tuscaniense* ISS-5309^T^, *C*. *ureicelerivorans* IMMIB RIV-2301^T^, and *C*. *pilbarense* IMMIB WACC-658^T^ (99.1–96.5%; Supplementary Table S2). Strain B5-R-101^T^ clustered with *C*. *aurimucosum*, strain TA-R-1^T^ with *C*. *aquatimens*, and strain BL-R-1^T^ with *C*. *mucifaciens* with strong bootstrap values supporting all the strains as a novel member of the genus *Corynebacterium* (Figure 1, Supplementary Figures S2, S3).

### 3.3. Genome analysis

The genome sizes of strains B5-R-101^T^, TA-R-1^T^, and BL-R-1^T^ were 2,644,957, 2,352,534, and 2,393,863 bp, respectively, and their G + C contents were 60.9, 66.1, and 65.6% (Table 2). The genome analysis of B5-R-101^T^ using RAST revealed a subsystem coverage of 29% across 703 SEED subsystems (678 non-hypothetical, 25 hypothetical). In contrast, strains TA-R-1^T^ and BL-R-1^T^ showed higher subsystem coverage, with 32 and 27% coverage, respectively, encompassing 689 and 607 subsystems. This coverage included 657 and 579 non-hypothetical subsystems, as well as 32 and 28 hypothetical subsystems for TA-R-1^T^ and BL-R-1^T^, respectively (Supplementary Figure S4). The distribution of functional groups revealed a predominant presence of genes associated with general processes, such as carbohydrates, amino acids and derivatives, and protein metabolism. Interestingly, the subsystems of B5-R-101^T^, TA-R-1^T^, and BL-R-1^T^ contained 101, 93, and 87 genes, respectively that are involved in synthesizing cofactors, vitamins, prosthetic groups, and pigments. These genes are responsible for the biosynthesis of B vitamins, including biotin, thiamin, pyridoxine, and folate, indicating that these strains can synthesize and transport B vitamins, making them highly desirable as probiotic strains (Rossi et al., 2011).

**Table 2:** Genomic features of strains B5-R-101^T^, TA-R-1^T^, and BL-R-1^T^.

Attributes	Features		
	B5-R-101^T^	TA-R-1^T^	BL-R-1^T^
Accessions	JAMFTR000000000	JAMFTQ000000000	JAPYJX000000000
Genomic size (bp)	2,644,957	2,352,634	2,393,863
G + C content (mol%)	60.9	66.1	65.6
No. of contigs	20	31	15
N50	241,478	133,040	278,662
Total genes	2,470	2,264	2,306
CDSs (Total)	2,403	2,200	2,243
Protein-coding gene	2,375	2,172	2,212
Genes (RNA)	67	64	63
rRNAs (5S, 16S, 23S)	4, 4, 4	3,3,3	4,3,2
tRANs	52	52	51
ncRANs	3	3	3
Pseudogenes	28	28	31
Genome coverage	150.0x	149.8x	148.4x
Phages	13	16	15
Resistance genes (CARD)	–	*ermX*	–

CDSs, coding sequences.

Genome analysis of strains B5-R-101^T^, TA-R-1^T^, and BL-R-1^T^ indicated probiotic potential in various genes associated with heat shock regulator (*hrcA*), molecular chaperones (*dna*G, *dna*J, *gro*EL, and *gro*ES), adhesion-related proteins (ClpB, ClpX, ClpS, and ClpC), and genes that confer resistance to acidic conditions (*csp*A). Moreover, theses strains contained genes that protect against osmotic stress (*opuA*, *opuC*, and *opuBD*) and have high adhesion ability (*tuf*, *lapA*, *srtA*1, *srtA*2, and *srtA3*) as well as vitamin biosynthesis genes (*copA*, *copZ*, *copZA*, and *csoR*) that may facilitate the reverse transport of vesicular proteins to the endoplasmic reticulum (Supplementary Table S3). Additionally, the genomic analysis also revealed the presence of multiple genes responsible for antioxidant activity, including genes related to oxidative stress, such as thioredoxin (*trx*), ferrous iron transporter (*feoB*, *oxyR*), glutathione (*cydA*, *yocS*, and *gpx*), NADH (*ndh*), and methionine sulfoxide reductase genes (*msrA*, *msrB*, and *msrC*; Supplementary Table S4). These genes are associated with antioxidant and probiotic potentials (Kandasamy et al., 2022). The CGView server created visual images of circular genomes that illustrate sequence characteristics, base composition plots, analysis outcomes, and sequences (Supplementary Figure S5). Genomic analysis revealed 44 phages across the three strains investigated. Specifically, B5-R-101^T^ contained 13 phages, TA-R-1^T^ contained 16, and BL-R-1^T^ contained 15. Additionally, we used the plasmid finder to test each strain for the presence of plasmid DNA but no plasmid replicons were detected. The novel strains B5-R-1^T^, TA-R-1^T^, and BL-R-1^T^, along with *C*. *aurimucosum* NRRL B-24143^T^, *C*. *aquatimens* IMMIB L-2475^T^, and *C*. *mucifaciens* DMMZ 2278^T^, showed a high degree of similarity, with more than 95% of the genome conserved between them when aligned with MAUVE alignment. This suggests that these species are closely related and have recent common ancestors. However, the MAUVE alignment also identified small, localized areas of the genome sequences that did not align with their closest neighbors, indicated by white vertical lines within blocks. These regions of conservation and variation suggest functional differences among the genomes (Supplementary Figure S6). The phylogenomic analysis conducted through genome-genome comparisons using TYGS server also supports strains B5-R-1^T^, TA-R-1^T^, and BL-R-1^T^ as the novel members of the genus *Corynebacterium* (Supplementary Figure S7). In addition, UBCG phylogenomic tree also supports all three strains as the novel members of the genus *Corynebacterium* (Supplementary Figure S8).

The genomic analysis of B5-R-101^T^, TA-R-1^T^, and BL-R-1^T^ showed that they have similar gene clusters that could produce antibiotic and antioxidant compounds, including terpene, polyketide, RiPP, saccharide, alkaloid, ectoine, NRP, and others (Supplementary Table S5). The COG functional groups of the genes in the strains were determined using EggNOG mapper v2, resulting in the categorization of 2,479, 2,228, and 2,275 genes, respectively. The distribution of functional groups was relatively equal among these three strains. The most significant active group was indicated as unknown, indicating its potential novelty and uniqueness (Supplementary Figure S9). Further analyses revealed the presence of loci that could be of interest, including proteins associated with phages, CRISPR, transport, and stress. The remaining proteins were classified into various functional groups, including those involved in transcription, RNA processing, chromatin structure, energy production, cell division, amino acid transport, replication, cell wall biogenesis, signal transduction, and more. The virulence Finder did not detect any virulence genes in the three strains. Furthermore, an *ermX* family gene was identified in TA-R-1^T^ using the antibiotic resistance database (CARD; Supplementary Figure S10). This gene shared 98.4% sequence identity with that of *C*. *glaucum* and displayed similar amino acid and nucleotide identities to the 23S rRNA methyltransferase [adenine (2503)-C (2); GenBank accession: AQQ15526]. However, no antibiotic-resistant genes were detected in the genome of strains B5-R-101^T^ and BL-R-1^T^.

### 3.4. Chemotaxonomic analysis

The principal fatty acids for all strains B5-R-101^T^, TA-R-1^T^, and BL-R-1^T^ were C_16:0_ and C_18:1_*ω*9*c*, similar major fatty acid profile that were obtained from other closest members of the genus *Corynebacterium* (Supplementary Table S6). Differential proportion of major fatty acids and presence or absence of minor fatty acids such as C_17:0_, C_18:0_, and C_13:1_ at 12–13, iso-C_17:0_, anteiso-C_11:0_, and hydroxy fatty acids differentiate strains B5-R-101^T^, TA-R-1^T^, and BL-R-1^T^ with other members of the genus *Corynebacterium* (Supplementary Table S6). The major polar lipids of strains B5-R-101^T^, and TA-R-1^T^ were diphosphatidylglycerol (DPG) and phosphatidylglycerol (PG). In addition, minor polar lipids such as an unidentified aminophosphoglycolipid, an unidentified aminophospholipid, an unidentified aminolipid, two unidentified glycopipids, and five unidentified polar lipids in strain B5-R-101^T^; an unidentified phosphoglycolipid, two unidentified aminolipids, and four unidentified polar lipids in strain TA-R-1^T^; and an unidentified aminophosphoglycolipid, two unidentified glycolipids, three unidentified aminolipids, and six unidentified polar lipids in strain BL-R-1^T^ were also detected (Supplementary Figure S11).

### 3.5. Hemolytic activity results

Hemolytic activity the three strains (B5-R-101^T^, TA-R-1^T^, and BL-R-1^T^) was negative (Supplementary Figure S12). The results obtained after 48 h of incubation at 37°C indicated that all the three strains could be utilized as probiotic safe candidates since the strains having negative hemolytic activities are regarded as safe for probiotics (Amoah et al., 2021; Gladysheva et al., 2023).

### 3.6. Survival under acidic conditions

The isolates were grown under various pH conditions to determine their ability to tolerate acidity in the stomach for probiotics potentiality. The pH test was conducted for 0–48 h at 37°C in BHI broth with a pH range of 2.0–6.0. The results showed that strains B5-R-101^T^, TA-R-1^T^, and BL-R-1^T^ exhibited tolerance to high acidic conditions (Supplementary Figure S13). Although their growth and survival rate gradually decreased after 30 h at pH 2.0–3.0, their growth remained stable after 10 h at pH 2.0–6.0.

### 3.7. Bile salt tolerance

Strains B5-R-101^T^, TA-R-1^T^, and BL-R-1^T^ were able to grow in a 0.3% bile salt concentration with survival rates ranging from 53.22 ± 1.48 to 62.96 ± 0.95% after 4 h of incubation (Table 3). B5-R-101^T^ and BL-R-1^T^ showed similar results, with survival rates of 62.16 ± 0.80 and 62.96 ± 0.95%, respectively, but TA-R-1^T^ exhibited a lower survival rate (53.22 ± 1.48%) than those of B5-R-101^T^ and BL-R-1^T^ at 0.3% bile salt concentrations.

**Table 3:** Tolerance of novel isolated *Corynebacterium* strains to 0.3 and 0.5% bile salt concentrations.

Strains	bile concentration (w/v; %)	Survival rate (%)			
		1 h	2 h	3 h	4 h
B5-R-101^T^	0.3	94.59 ± 0.80	83.78 ± 01.10	72.97 ± 1.78	62.16 ± 0.80
	0.5	4.05 ± 0.53	0.14 ± 0.05	0 ± 00	0 ± 00
TA-R-1^T^	0.3	80.64 ± 1.48	61.29 ± 2.62	59.03 ± 4.8	53.22 ± 1.48
	0.5	82.25 ± 1.02	56.45 ± 1.2	0.03 ± 0.12	0 ± 00
BL-R-1^T^	0.3	100 ± 1.02	85.81 ± 3.12	70.37 ± 1.02	62.96 ± 0.95
	0.5	39.5 ± 0.56	37.03 ± 1.9	0 ± 00	0 ± 00

### 3.8. Antimicrobial activity

**Figure 2:**
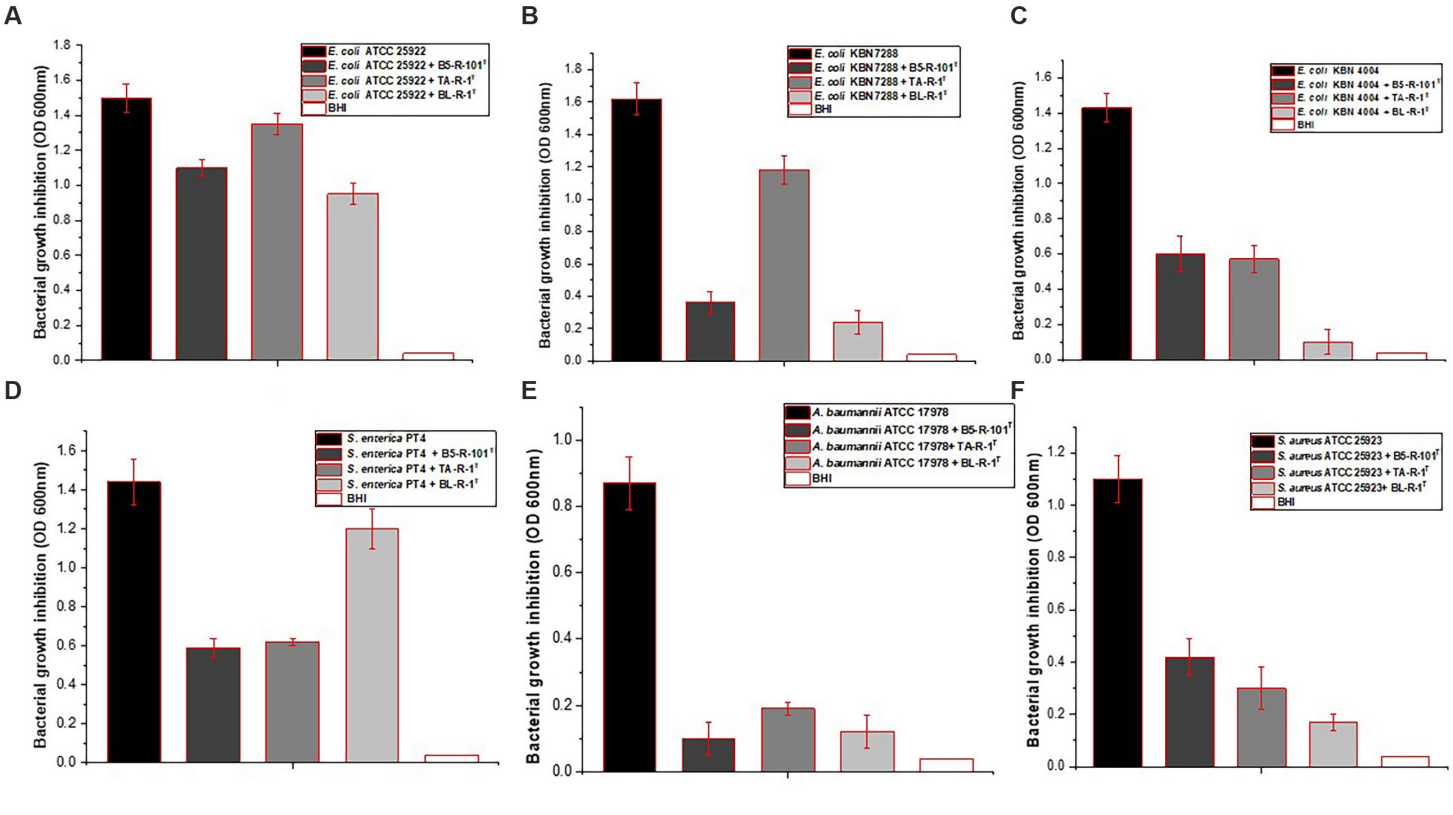
The antimicrobial activity of strains B5-R-101^T^, TA-R-1^T^, and BL-R-1^T^ against four different pathogens, including *Escherichia coli*, *Salmonella enterica*, *Acinetobacter baumannii*, and *Staphylococcus aureus*.

The study investigated the antimicrobial activity of the three isolated strains against four different pathogens, including *E*. *coli*, *A*. *baumannii*, *Salmonella*, and *S*. *aureus*. Strains B5-R-101^T^, TA-R-1^T^, and BL-R-1^T^ had inhibitory effects on the selected pathogens (Figure 2). Strain B5-R-101^T^ showed relatively low inhibitory activity against *E*. *coli* ATCC 25922, whereas TA-R-1^T^ and BL-R-1^T^ showed low inhibitory activity against *E*. *coli* ATCC 25922, *E*. *coli* KBN 07288, and *E*. *coli* KBN 04004. All three isolates significantly inhibited *A*. *baumannii* ATCC 17978, *S*. *enterica* PT4, and *S*. *aureus* ATCC 25923.

### 3.9. Scavenging activity on 2,2-diphenyl-1-picrylhydrazyl radicals

**Figure 3:**
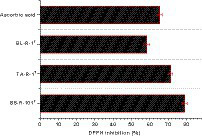
2,2-Diphenyl-1-picrylhydrazyl (DPPH) radical scavenging activities of strains B5-R-101^T^, TA-R-1^T^, BL-R-1^T^, and ascorbic acid (vitamin C). Data are given as mean ± SEM.

Strains B5-R-101^T^, TA-R-1^T^, and Bl-R-1^T^ exhibited high antioxidant activity, with scavenging activity ranging from 58 ± 1.62 to 79 ± 1.46% (Figure 3). The bacterial strain B5-R-101^T^ showed the highest antioxidant activity at 79 ± 1.46%, followed by TA-R-1^T^ at 71.4 ± 1.03% and BL-R-1^T^ at 58.2 ± 1.62%. In the control group, an ascorbic acid concentration of 6 μg/mL demonstrated the highest antioxidant activity at 65.42 ± 1.31%.

### 3.10. Evaluation of the antibiotic susceptibility

Strain B5-R-101^T^ was susceptible to most antibiotics among the 12 antibiogram disks, except for colistin (COL), aztreonam (ATM), and oxacillin (OXA). Strains TA-R-1^T^ and BL-R-1^T^ were both resistant to erythromycin (ERY), colistin (COL), and aztreonam (ATM). TA-R-1^T^ appeared moderately susceptible to ciprofloxacin (CIP) and oxacillin (OXA), whereas BL-R-1^T^ was moderately susceptible to ciprofloxacin (CIP). The results were interpreted based on the Clinical and Laboratory Standards Institute (CLSI) guidelines (CLSI, 2018; Table 4).

**Table 4:** Antibiotic susceptibility test results of the novel isolated *Corynebacterium* strains on Brain heart infusion agar (BHA).

Name of the antibiotics	B5-R-101^T^	TA-R-1^T^	BL-R-1^T^
Ampicillin (AMP)	S	S	S
Gentamicin (GEN)	S	S	S
Ceftazidime (CAZ)	S	S	S
Erythromycin (ERY)	S	R	R
Piperacillin (PIP)	S	S	S
Cefoxitin (FOX)	S	S	S
Colistin (COL)	R	R	R
Cefotaxime (CTZ)	S	S	S
Amikacin (AMK)	S	S	S
Aztreonam (ATM)	R	R	R
Ciprofloxacin (CIP)	S	I	I
Oxacillin (OXA)	R	I	S

R, resistant; I, intermediate; and S, susceptible. The interpretive zones for each antibiotic were assigned according to Charteris et al. (1998).

## 4. Discussion

This study has isolated three novel species of the genus *Corynebacterium* designated as B5-R-101^T^, TA-R-1^T^, and BL-R-1^T^. These strains have significant probiotic properties and exhibit remarkable antioxidant and antimicrobial activities. Phylogenetic analysis based on the 16S rRNA gene sequence showed that these species belonged to the genus *Corynebacterium*, with a sequence similarity of 98.5–99.0% with other members of the same genus. Additionally, ANI and dDDH values confirmed that these strains were distinct from previously described species and represent the novel species within the genus *Corynebacterium* (Supplementary Table S7).

This study evaluated the antioxidant properties of strains B5-R-101^T^, TA-R-1^T^, and BL-R-1^T^ by using the DPPH radical scavenging assay. Genome analysis also revealed that these strains contained multiple genes responsible for antioxidant activity, including genes related to oxidative stress such as thioredoxin (*trx*), ferrous iron transporter (*feoB* and *oxyR*), glutathione (*cydA*, *yocS*, and *gpx*), and NADH (*ndh*). The thioredoxin system and glutathione systems remove ROS and RNS and the ferrous iron transporter regulates iron levels. NADH oxidase/peroxidase and catalase degrade hydrogen peroxide and ROS directly (Kandasamy et al., 2022). Additionally, these strains possessed genes for catalase (*katE*), pyruvate oxidase (*pox*, *tpxD*), and glutaredoxin (*nrdH*), which are reported to involved in regulating iron levels and repairing oxidized methionine residues caused by ROS (Kong et al., 2019; Gao et al., 2020; Kandasamy et al., 2022). These strains also have superoxide dismutase and genes coding for a manganese transport system (*mntA*, *mntB*, and *mntC*) and a protein (MntR) that accumulate Mn^2+^ to counteract oxidative stress. Furthermore, the strains contain methionine sulfoxide reductase genes (*msrA*, *msrB*, and *msrC*), which facilitate the repair of oxidized methionine residues induced by ROS in proteins (Gao et al., 2020; Li et al., 2021). Strain B5-R-101^T^ contains the genes *orN* and *glo*1, whereas TA-R-1^T^ and BL-R-1^T^ contain *sufC*, *sufD*, *sufR*, and *sufE*2, which are involved in neutralizing ROS and protecting against oxidative stress (Distler and Palmer, 2012). Similarly, the presence of genes in the genome of these three strains such as, *ahpC*, *ahpD*, and *ahpF* help to detoxify harmful compounds (Teramoto et al., 2013). Overall, the presence of genes related to antioxidant activities in the genome of these strains confer enhanced oxidative stress tolerance and enable them to adapt to various environments.

The CFSs of the isolates were analyzed to determine their antimicrobial properties. All the three strains showed strong antibacterial activity against four multidrug resistant bacteria, including three different *E*. *coli* strains. Probiotic strains fight infections by producing antimicrobial substances that prevent the growth of harmful bacteria in the digestive tract. The secondary metabolites found in the three isolates (nonalpha poly amino acids, indigoidine, fellutamide B, terpenes, polyketides, and RiPPs may have good antimicrobial, antiviral, and anti-inflammatory properties, and analgesic effects; Zhang et al., 2020).

Genomic analysis revealed that the strains B5-R-101^T^, TA-R-1^T^, and BL-R-1^T^ possess an abundance of genes that promote gut health, allowing probiotics to thrive and provide numerous benefits for overall wellbeing. The genes identified in this analysis included a heat shock regulator (*hrcA*), molecular chaperones (*dnaG*, *dnaJ*, *groEL*, and *groES*), protease-coding genes (*clpB*, *clpX*, *clpS*, and *clpC*), and a cold shock protein-coding gene (*cspA*). The CSP family is particularly important for maintaining gut health due to its role in regulating protein aggregation, stabilizing membranes, and enhancing resistance to extreme temperatures (Li et al., 2021). The genomic analysis also revealed the presence of genes that confer resistance to acidic conditions, including clusters of F0F1-ATP synthase subunits essential for regulating acid tolerance in the cytoplasm (Sun et al., 2022). Additionally, we found genes encoding sodium-proton (Na+/H+) antiporters (*nhaC*), an alkaline shock protein (*asp*23), and a glutamate decarboxylase (*gadB*) that play important roles in pH and Na + homeostasis. Moreover, the genome analysis detected *ppaC*, which encodes for inorganic pyrophosphatase that maintains surface tension, and *cfa*, which encodes for cyclopropane-fatty-acyl-phospholipid that enhances lipid synthesis. These genes (*ppaC* and *cfa*) are crucial for maintaining the integrity of the cell membrane and enhancing lipid synthesis, respectively (Li et al., 2021). We also detected *opuA*, *opuC*, and *opuBD*, which protect against osmotic stress and confer high adhesion ability due to their cell surface proteins (Kandasamy et al., 2022). The strains also contained adhesion-related proteins, such as signal peptidase II (*lspA*), elongation factor Tu (*tuf*), sortase A (*srtA*1, *srtA*2, and *srtA*3), LPXTG motif, and glycosyltransferase (*epsH*), indicating a high degree of adhesion (Sun et al., 2022). Genes with quorum sensing functions (*luxS* and *luxR*) contribute to the survival and production of valuable molecules (Ball et al., 2017). Additionally, *fth*1 maintains gut iron homeostasis and is involved in oxidative stress tolerance (Arosio and Levi, 2002). Strains B5-R-101^T^ and TA-R-1^T^ also contain *mutT*, *mutT*2, and *mutT*3 genes, which protect probiotics from stress and maintain genome stability (Da Silva et al., 2019). Strains TA-R-1^T^ and BL-R-1^T^ contain *yidC*, which is involved in various cellular functions such as protein folding, membrane integrity, signaling, molecule transport, energy metabolism, and possibly antibiotic resistance. Moreover, vitamin biosynthesis genes, including *copA*, *cop*Z, *copZA*, and *csoR* detected in BL-R-1^T^, may facilitate the reverse transport of vesicular proteins to the endoplasmic reticulum (Zeng et al., 2021).

The ability to withstand low pH and bile salts is crucial for bacteria to survive and grow in the gastrointestinal tract. The resistance to low pH of the gastric juice in the abdomen and the bile salt in the bowel is the major selection criteria for probiotic (Zhang et al., 2020; Amoah et al., 2021). All the three strains used in this study could survive in the lower pH and in 0.3% bile salt for 4 h, indicating these strains as a potential probiotic candidate.

The genome analysis of the isolates revealed that they did not contain any genes linked to pathogenicity or virulence. This study also confirmed the safety of the probiotic strains since none showed hemolytic activity. In previous studies, several *Corynebacterium* species, including *C*. *accolens*, *C*. *glutamicum*, *C*. *lactis*, and *C*. *amycolatum*, none of which exhibited hemolytic activity, were found to be potential probiotic species (Teramoto et al., 2013; Gladysheva et al., 2023). The genome of strains B5-R-101^T^ and BL-R-1^T^ contained no antibiotic resistance genes. However, antibiotic resistance gene, *ermX*, was detected in the genome of strain TA-R-1^T^, which encodes an enzyme capable of modifying the DNA sequence of the 23S rRNA gene through methylation, resulting in resistance to macrolides, lincosamides, and streptogramin B (Chen et al., 2007). A previous study analyzing the whole genomes of 126 probiotics uncovered several resistance genes on plasmids, which can be transferred to other bacteria (Fatahi-Bafghi et al., 2022). However, we did not find plasmids carrying the transferred gene in the genome of the strain TA-R-1^T^. Therefore, TA-R-1^T^ could be considered for Quality Presumption of Safety (QPS) status.

## 5. Conclusion

This study provides exciting insights into the potential probiotic benefits of three novel species of the genus *Corynebacterium*. The genomic analysis of these strains showed promising characteristics that make them strong candidates for probiotics. Specifically, the strains possess genes related to adhesion, resistance to high acidity, and vitamin synthesis, all of which are essential probiotic properties. The ability of the strains to survive and grow under high bile salt concentrations is particularly noteworthy as it indicates their potential efficacy as probiotics. Additionally, the strains’ inability to produce harmful toxins or enzymes indicate their safety as potential probiotic candidates. These findings suggest that strains B5-R-101^T^, TA-R-1^T^, and BL-R-1^T^ could be valuable for probiotic applications, particularly for improving gut health. However, further studies are needed to investigate the strains’ efficacy and safety *in vivo* and elucidate their mechanisms of action. Overall, this study highlights the potential of the novel *Corynebacterium* species as a valuable addition to the probiotic arsenal. With input from further research, these strains may be effective probiotics that can benefit human health.

## 6. Species protologue

### 6.1. Description of *Corynebacterium intestinale* sp. nov.

*Corynebacterium intestinale* (in.tes.ti.na’le. N.L. neut. adj. *intestinale*, pertaining to the intestine).

Cells are facultatively anaerobic, Gram-stain-positive, non-motile, and rod-shaped. Colonies are slightly yellow and sticky in appearance. Cells grow well on BHA with 5% defibrinated sheep blood and weakly on LBA and MHA. Growth temperature ranges from 10 to 42°C (optimum, 32–37°C) and pH 2.0–10.0 (optimum, 4.5–8.0). Cells can tolerate NaCl concentrations of up to 10.5% (w/v) and optimally grow at 0.5–1.5% NaCl. Catalase is positive while oxidase test is negative. Gelatin and aesculin are hydrolysed but tyrosine, casein, starch, Tween 20, Tween 40, and Tween 80 are not. Glucose is fermented and negative for urease activity. The type strain shows the following enzyme activities: positive for alkaline phosphatase, esterase (C4), esterase lipase (C8), lipase (C14), leucine arylamidase, valine arylamidase, cystine arylamidase, trypsin, *α*-chymotrypsin, acid phosphatase, and naphthol-AS-BI-phosphohydrolase; weakly positive for *α*-galactosidase; and negative for *β-*galactosidase, *β*-glucuronidase, *α*-glucosidase, *β*-glucosidase, *N*-acetyl-*β*-glucosaminidase, *α*-mannosidase, and *α*-fucosidase. Cells weakly assimilate d-glucose, d-fructose, d-mannose, d-maltose, arbutin, sucrose, and 2-ketogluconate. The principal cellular fatty acids are C_18:1_*ω*9*c*, C_16:0_, iso-C_15:0_ 3-OH, C_13:1_ at 12–13, and anteiso-C_11:0_. The major polar lipids are diphosphatidylglycerol, phosphatidylglycerol, and an unidentified aminophospholipid. The DNA G + C content of the type strain is 60.9%.

The type strain, B5-R-101^T^ (= CGMCC 1.19408^T^ = KCTC 49761^T^), was isolated from human feces in the Department of Microbiology at Kyungpook National University, Republic of Korea. The GenBank/EMBL/DDNJ accession numbers for the 16S rRNA gene sequence and the whole-genome sequence of strain B5-R-101^T^ are OK147962 and JAMFTR00000000, respectively.

### 6.2. Description of *Corynebacterium stercoris* sp. nov.

*Corynebacterium stercoris* (ster’co.ris. L. gen. n. *stercoris* of feces, referring to the source of isolation).

Cells, are Gram-stain-positive, facultative anaerobic, non-motile, grow well on BHA with 5% defibrinated sheep blood and weakly grow on LBA and MHA. After 48 h of incubation at 37°C, colonies are circular, dry, and gray with a diameter of 1.0 mm. Cells grow well at temperatures ranging from 10 to 45°C (optimum, 32–37°C), at pH levels of 2.0–10.0 (optimum, 5.0–8.5), and tolerate NaCl concentrations of up to 9.5% (w/v). Catalase activity is positive and oxidase is negative. Glucose is fermented. Aesculin and gelatin are hydrolyzed but not starch, casein, tyrosine, Tween 20, Tween 40, and Tween 80. Nitrate is not reduced to nitrite. The type strain exhibits the following enzyme activities: positive for alkaline phosphatase, esterase (C4), esterase lipase (C8), lipase (C14), leucine arylamidase, valine arylamidase, cystine arylamidase, trypsin, *α*-chymotrypsin, acid phosphatase, naphthol-AS-BI-phosphohydrolase, and *β*-glucosidase; and negative for *β*-glucuronidase, *α*-glucosidase, *N*-acetyl-*β*-glucosaminidase, *α*-mannosidase, and *α*-fucosidase. Glucose is fermented. d-glucose, d-fructose, d-mannose, d-maltose, sucrose, 2-ketogluconate, and arbutin are weakly assimilated. The major cellular fatty acids are C_16:0_, C_18:1_*ω*9*c*, anteiso-C_19:0_, and C_18:0_ 10-methyl. The principal polar lipids are diphosphatidylglycerol, phosphatidylglycerol, and an unidentified phosphoglycolipid. The DNA G + C content of type strain is 66.1%.

This type strain, TA-R-1^T^ (=CGMCC 1.60014^T^ = KCTC 49742^T^), was isolated from human feces in the Department of Microbiology at Kyungpook National University, Republic of Korea. The GenBank accession numbers for the 16S rRNA sequence and the whole genome sequence of strain TA-R-1^T^ are OK148071 and JAMFTQ000000000, respectively.

### 6.3. Description of *Corynebacterium faecium* sp. nov.

*Corynebacterium faecium* (fae’ci.um. L. gen. pl. n. *faecium*, of the dregs, of feces).

Cells are Gram-stain-positive, facultative anaerobic, non-motile, circular, and formed yellowish colonies with 1–1.5 mm diameter after 48 h of incubation on BHA with 5% defibrinated sheep blood at 37°C. Grow well on blood agar but weakly on MHA and LBA. Cells grow well at 10–42°C (optimum, 32–37°C), at pH 2.0–9.0 (optimum, 5.0–7.5) and can tolerate NaCl concentrations up to 10.5%. Urease activity is positive. Hydrolyzed aesculin and gelatin but not casein, starch, tyrosine, Tween 20, Tween 40, and Tween 80. Nitrate is not reduced to nitrate. The type strain exhibits the following enzyme activities: positive for alkaline phosphatase, esterase (C4), esterase lipase (C8), lipase (C14), leucine arylamidase, valine arylamidase, cystine arylamidase, acid phosphatase, naphthol-AS-BI-phosphate, and *α*-galactosidase; and negative for *β-*galactosidase, *β*-glucuronidase, *α*-glucosidase, *β*-glucosidase, *N*-acetyl-*β*-glucosaminidase, *α*-mannosidase, and *α*-fucosidase. Acid is produced from 2-ketogluconate, gluconate, sucrose, d-maltose, l-rhamnose, d-fructose, d-glucose, d-adonitol, d-ribose, and l-arabinose. The principal cellular fatty acids are C_18:1_*ω*9*c*, C_16:0_, and C_18:0_ whereas the major polar lipids are diphosphatidylglycerol, phosphatidylglycerol, and an unidentified aminophospholipid. The DNA G + C content of type strain is 65.6%.

The type strain, BL-R-1^T^ (= KCTC 49735^T^ = TBRC 17331^T^), was isolated from the human feces in Department of Microbiology at Kyungpook National University, Republic of Korea. The GenBank accession numbers for the 16S rRNA sequence and the whole-genome sequence of strain BL-R-1^T^ are OK147994 and JAPYJX000000000, respectively.

## Data availability statement

The datasets presented in this study can be found in online repositories. The names of the repository/repositories and accession number(s) can be found in the article/Supplementary material.

## Ethics statement

The studies involving human participants were reviewed and approved by Institutional Review Board of Kyungpook National University Hospital (KNUH 2021-03-011-002). The patients/participants provided their written informed consent to participate in this study.

## Author contributions

RD conceived and designed the experiments. MS and RD conducted all the experiments. SK interpreted the data. JK coordinated and supervised this study. MS, RD, SK, and JK analyzed the data and prepared the manuscript. All authors contributed to the article and approved the submitted version.

## Funding

This research was supported by the Basic Science Research Program through the National Research Foundation of Korea (NRF) funded by the Ministry of Education (grant number NRF-2017R1D1A3B06032486).

## Conflict of interest

The authors declare that the research was conducted in the absence of any commercial or financial relationships that could be construed as a potential conflict of interest.

## Publisher’s note

All claims expressed in this article are solely those of the authors and do not necessarily represent those of their affiliated organizations, or those of the publisher, the editors and the reviewers. Any product that may be evaluated in this article, or claim that may be made by its manufacturer, is not guaranteed or endorsed by the publisher.

## Footnotes

^1^ https://www.arb-silva.de/aligner/

^2^ https://www.ezbiocloud.net/tools/contest16s

^3^ https://rast.nmpdr.org

^4^ https://www.ncbi.nlm.nih.gov/genome/annotation_prok

^5^ https://darlinglab.org/mauve/mauve.html

^6^ https://proksee.ca/

^7^ https://card.mcmaster.ca/

^8^ https://antismash.secondarymetabolites.org/

^9^ https://www.ezbiocloud.net/tools/orthoani

^10^ https://tygs.dsmz.de/
