# Supplementary material for: Genome insight and probiotic potential of three novel species of the genus Corynebacterium
Source: Front Microbiol. 2023 Jul 6;14:1225282. doi: 10.3389/fmicb.2023.1225282 (PMC10358988; doi:10.3389/fmicb.2023.1225282)
Supplement: Supplementary file 1 [file Data_Sheet_1.pdf]

Genome insight and probiotic potential of three novel species of the genus *Corynebacterium*

†Md Shamsuzzaman[1], †Ram Hari Dahal[2], Shukho Kim[1,2], and Jungmin Kim[1,2]*

[1]Department of Biomedical Science, School of Medicine, Kyungpook National University, Daegu 41944, Republic of Korea

[2]Department of Microbiology, School of Medicine, Kyungpook National University, Daegu 41944, Republic of Korea

Running title

Antioxidant, antimicrobial, and probiotic potential

†These authors contributed equally.

***Correspondence:**
Jungmin Kim
Phone: +82-53-420-4840; Fax: +82-53-427-5664; E-mail: minkim@knu.ac.kr

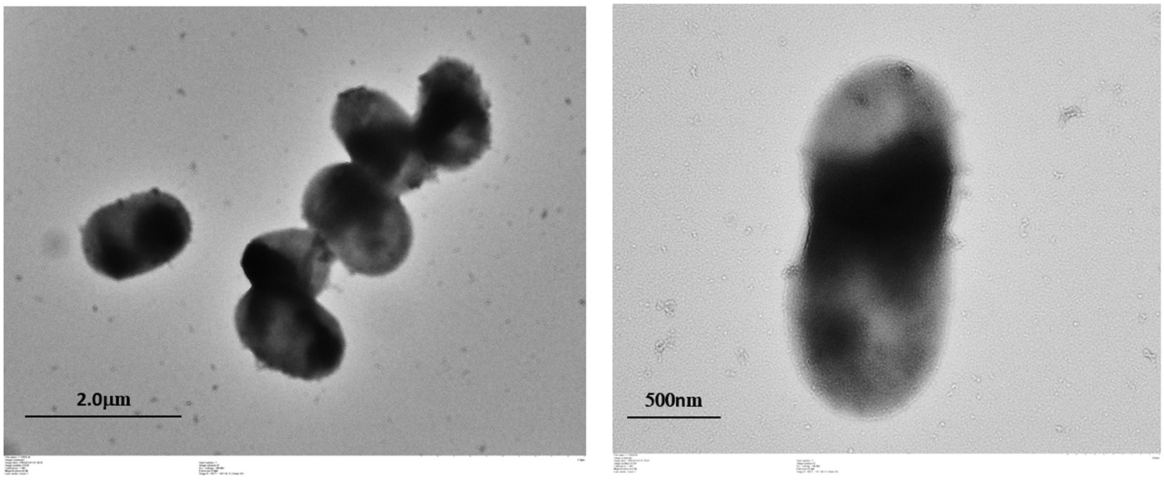

(a)

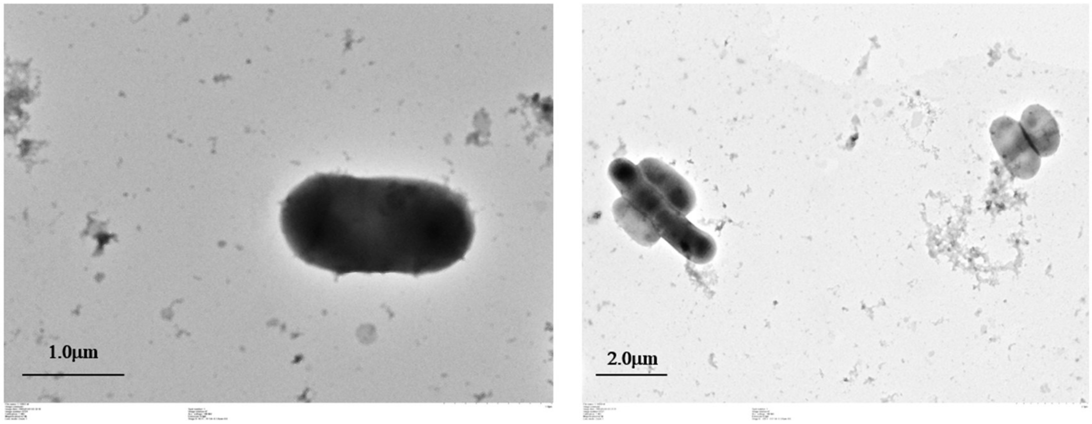

(b)

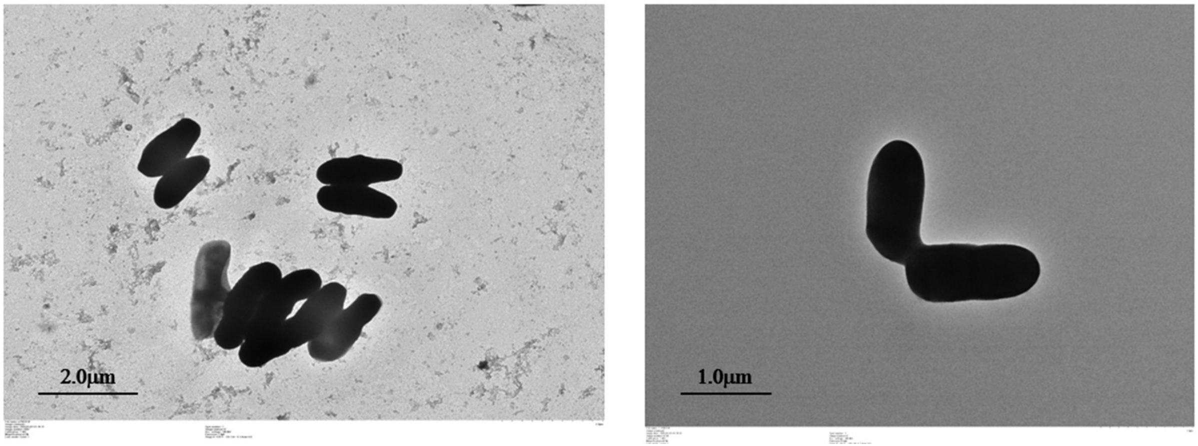

(c)

Supplementary Figure S1. Transmission electron microscopic image of strain (a) B5-R-101^{T}, (b) TA-R-1^{T} and (c) BL-R-1^{T} grown on 5% sheep blood agar at 37 °C for 24-48 hours.

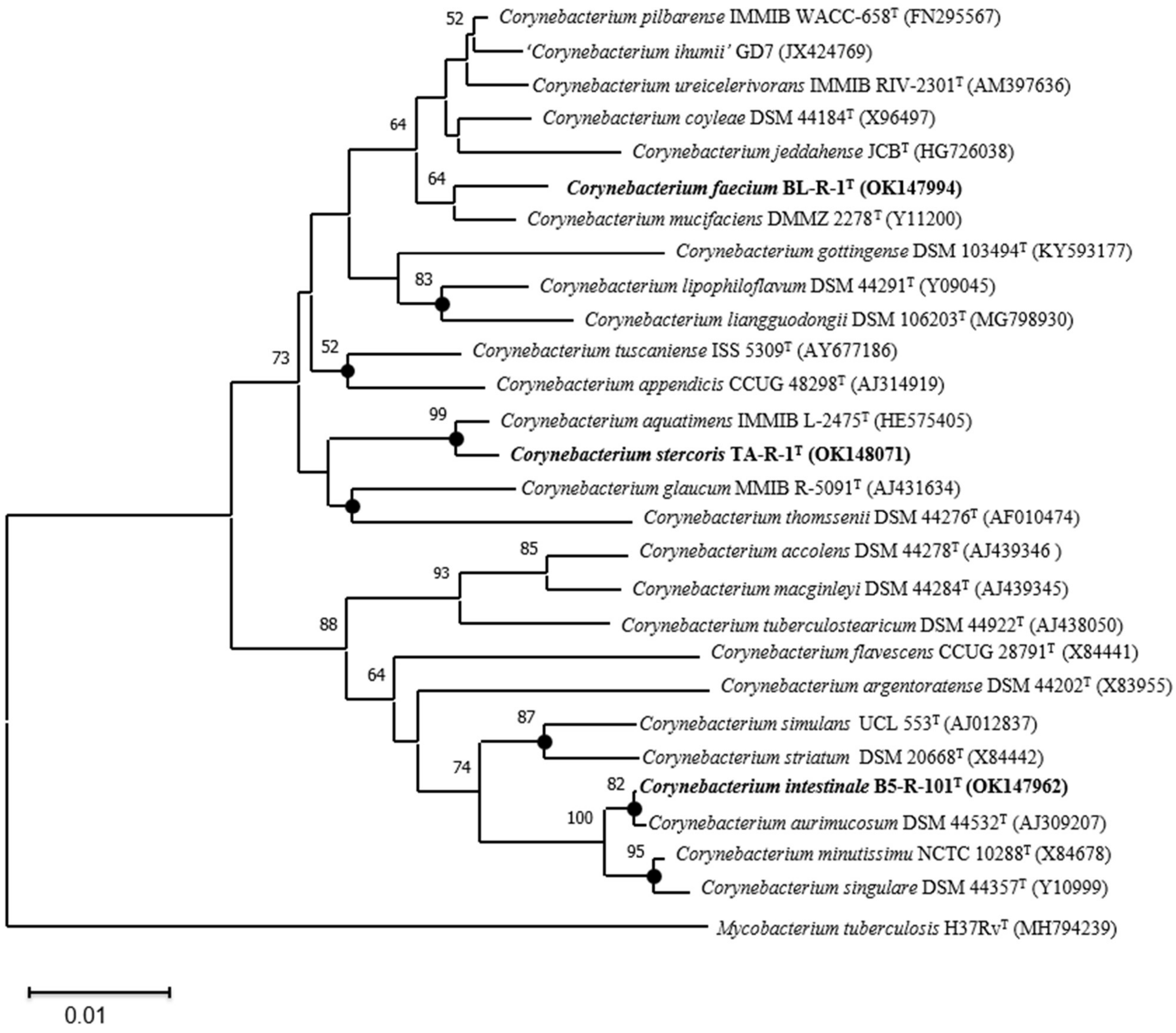

Supplementary Figure S2. The neighbour-joining (NJ) tree reconstructed using the almost complete 16S rRNA gene sequences to display the phylogenetic position of the strains B5-R-101^{T}, TA-R-1^{T,} and BL-R-1^{T} to their closely related members of the genus *Corynebacterium*. Nodes consistently recovered by all three treeing methods (neighbor-joining, maximum-likelihood, and maximum-parsimony) are represented by filled circles. The percentage of 1000 bootstrap replicates are indicated by node numbers, with only values greater than or equal to 50% displayed. The out-group used was *Mycobacterium tuberculosis* H37Rv^{T}. Nucleotide accession numbers are given in parentheses. The bar represents 0.01 substitutions per nucleotide position.

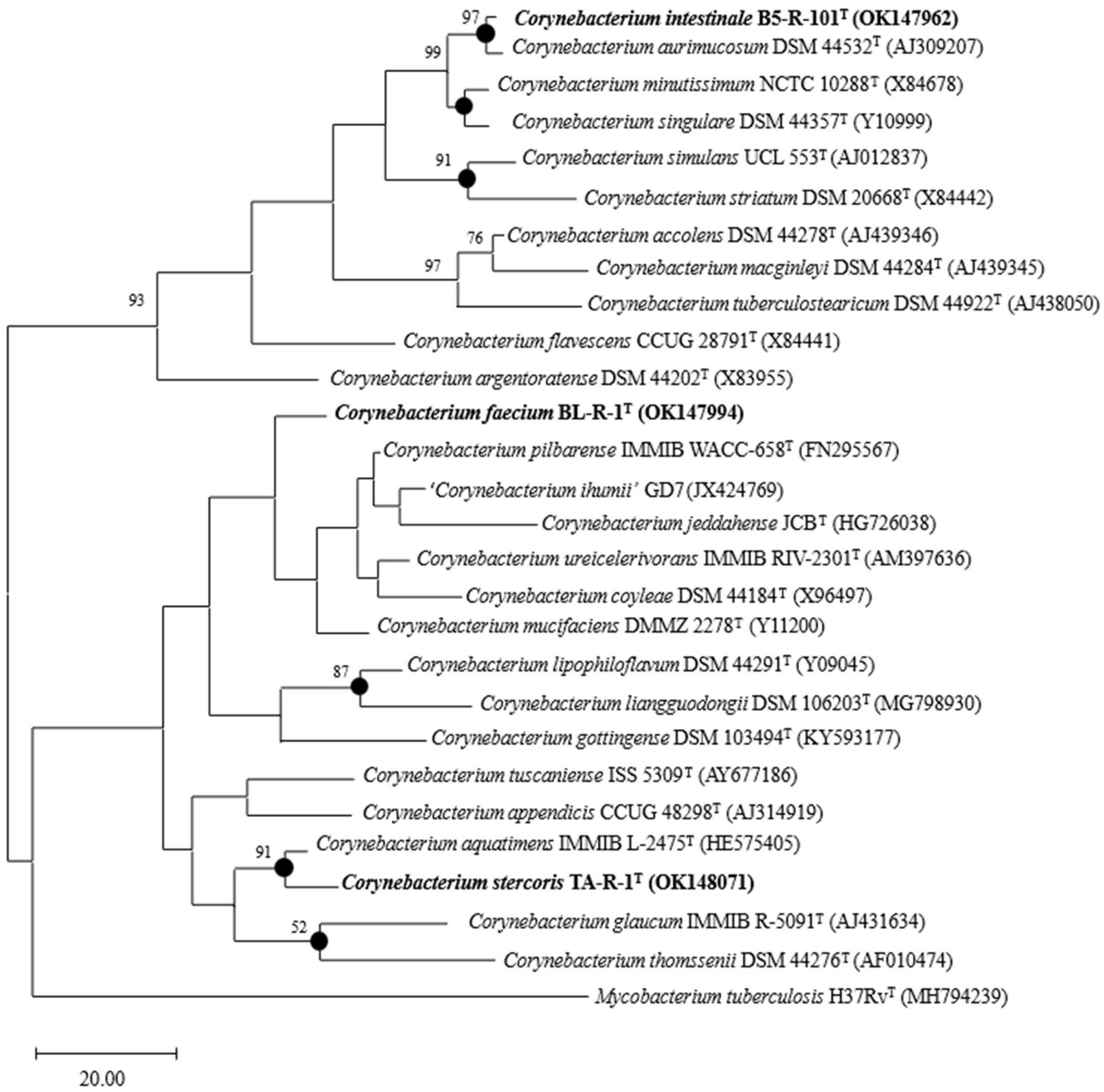

Supplementary Figure S3. The maximum–parsimony tree reconstructed using the almost complete 16S rRNA gene sequences to display the phylogenetic position of the strains B5-R-101^T, TA-R-1^{T,} and BL-R-1^T to their closely related members of the genus *Corynebacterium*. Nodes consistently recovered by all three treeing methods (neighbor-joining, maximum-likelihood, and maximum-parsimony) are represented by filled circles. The percentage of 1000 bootstrap replicates are indicated by node numbers, with only values greater than or equal to 50% displayed. The out-group used was *Mycobacterium tuberculosis* H37Rv^T. Nucleotide accession numbers are given in parentheses. The bar represents 0.01 substitutions per nucleotide position.

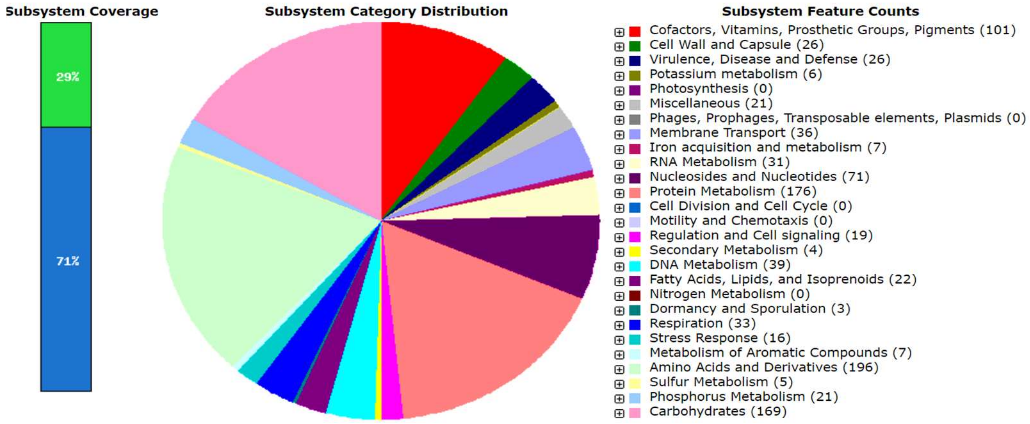

Supplementary Figure S4 (a). The subsystem feature counts for strain B5-R-101^{T} analysed by RAST (Rapid Annotation using Subsystem technology).

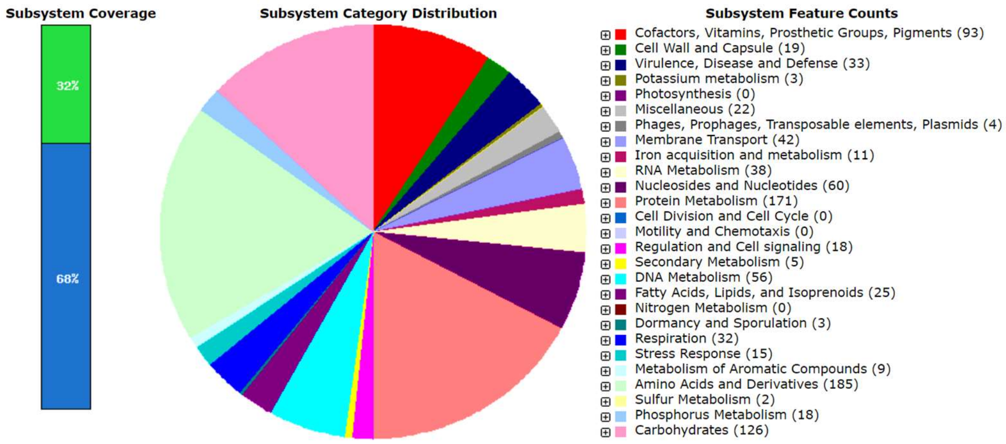

Supplementary Figure S4 (b). The subsystem feature counts for strain TA-R-1^{T} analysed by RAST (Rapid Annotation using Subsystem technology).

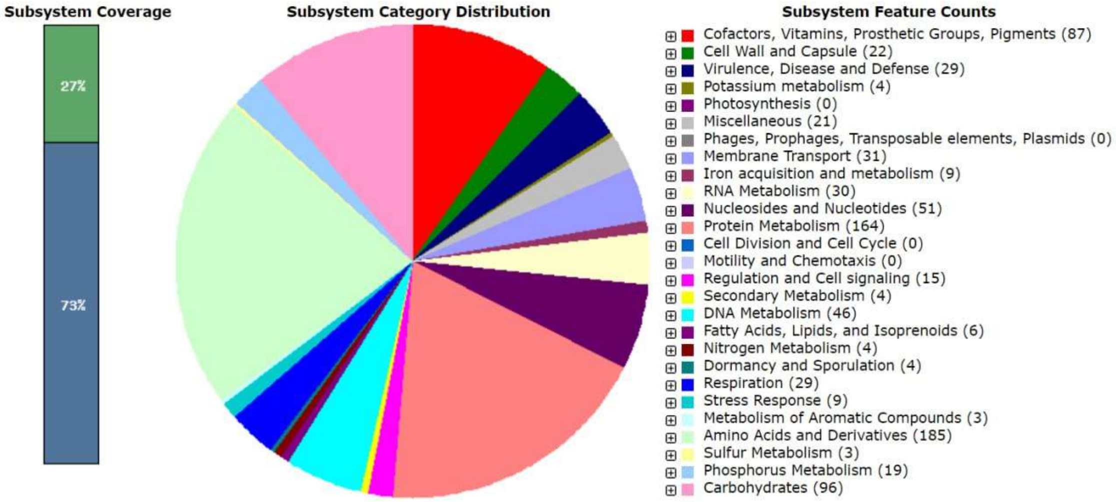

Supplementary Figure S4 (c). The subsystem feature counts for strain BL-R-1^T analysed by RAST (Rapid Annotation using Subsystem technology).

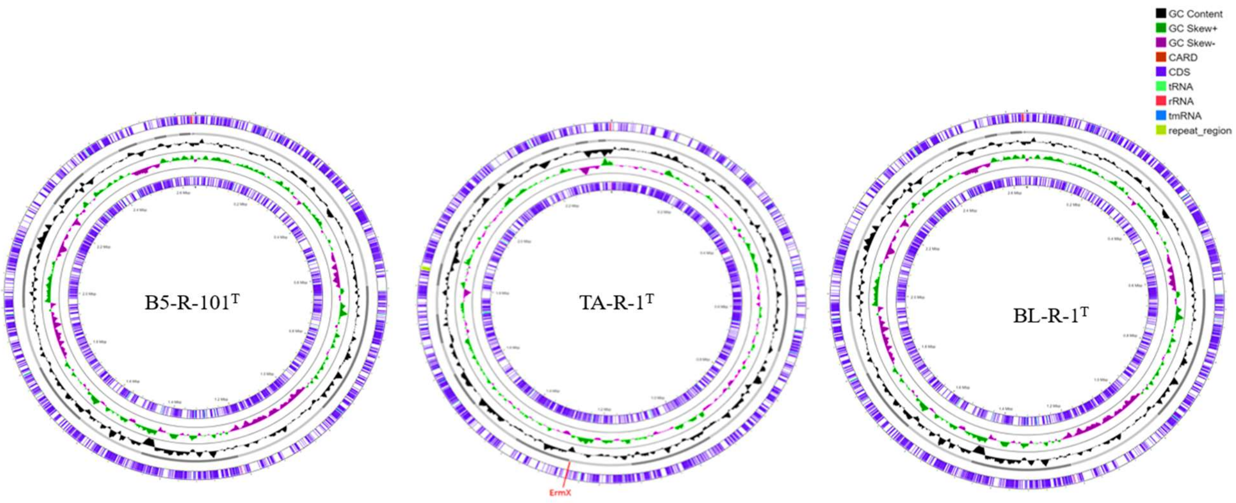

Supplementary Figure S5. The figure shows a graphical circular representation of three chromosomes of strains B5-R-101^T, TA-R-1^T, and BL-R-1^T. The figure displays various genomic features such as CDS on the forward and reverses strands, tRNA, rRNA, GC content, and GC skew, marked from the edge to the center. The red pane in TA-R-1^T highlights the *ermX*-resistant gene.

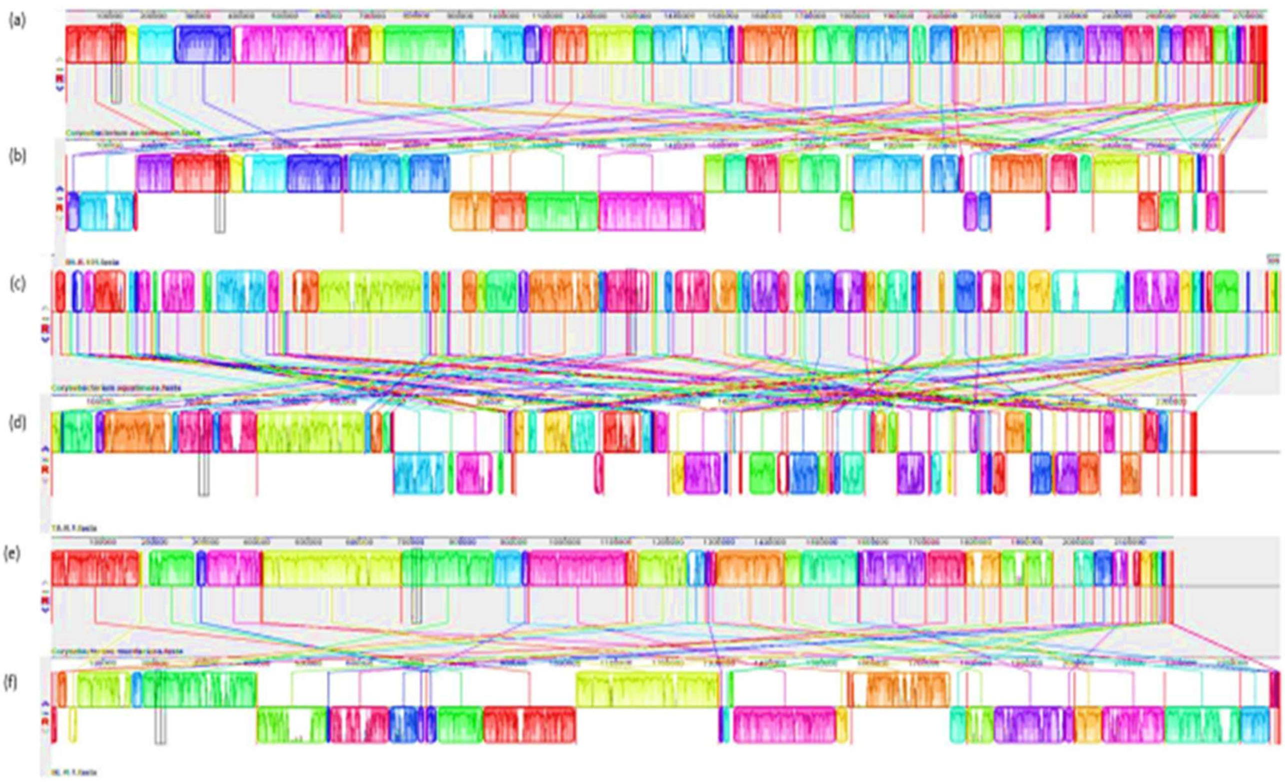

Supplementary Figures S6. In this figure, we employed the progressive Mauve algorithm (v2.3.1) to perform multiple alignments of the genomes of several *Corynebacterium* strains. The objective was to investigate the rearrangement patterns and synteny in these genomes. The genomes were laid out horizontally, with homologous segments represented by coloured blocks. Blocks that were shifted downward relative to the closest genome defined reversed segments. The regions outside the blocks lacked homology and were represented by white areas, which were unique to each genome and not aligned. The similarity profiles provided an estimate of the degree of sequence conservation. These results indicate that B5-R-101^{T}, TA-R-1^{T}, and BL-R-1^{T} exhibited high sequence variability, as evidenced by their genome's plethora of white areas. These observations suggest that the genomes of these strains have undergone significant genomic changes. On the other hand, compared with their closest neighbours showed a high degree of similarity. We also found many white vertical lines that indicated they were not similar. (a) *C. aurimucosum* IMMIB D-1488^{T}, (b) B5-R-101^{T}, (c) *C. aquatimens* IMMIB L-2475^{T}, (d) TA-R-1^{T}, (e) *C. mucifaciens* DMMZ 2278 ^{T} and (f), BL-R-1^{T}.

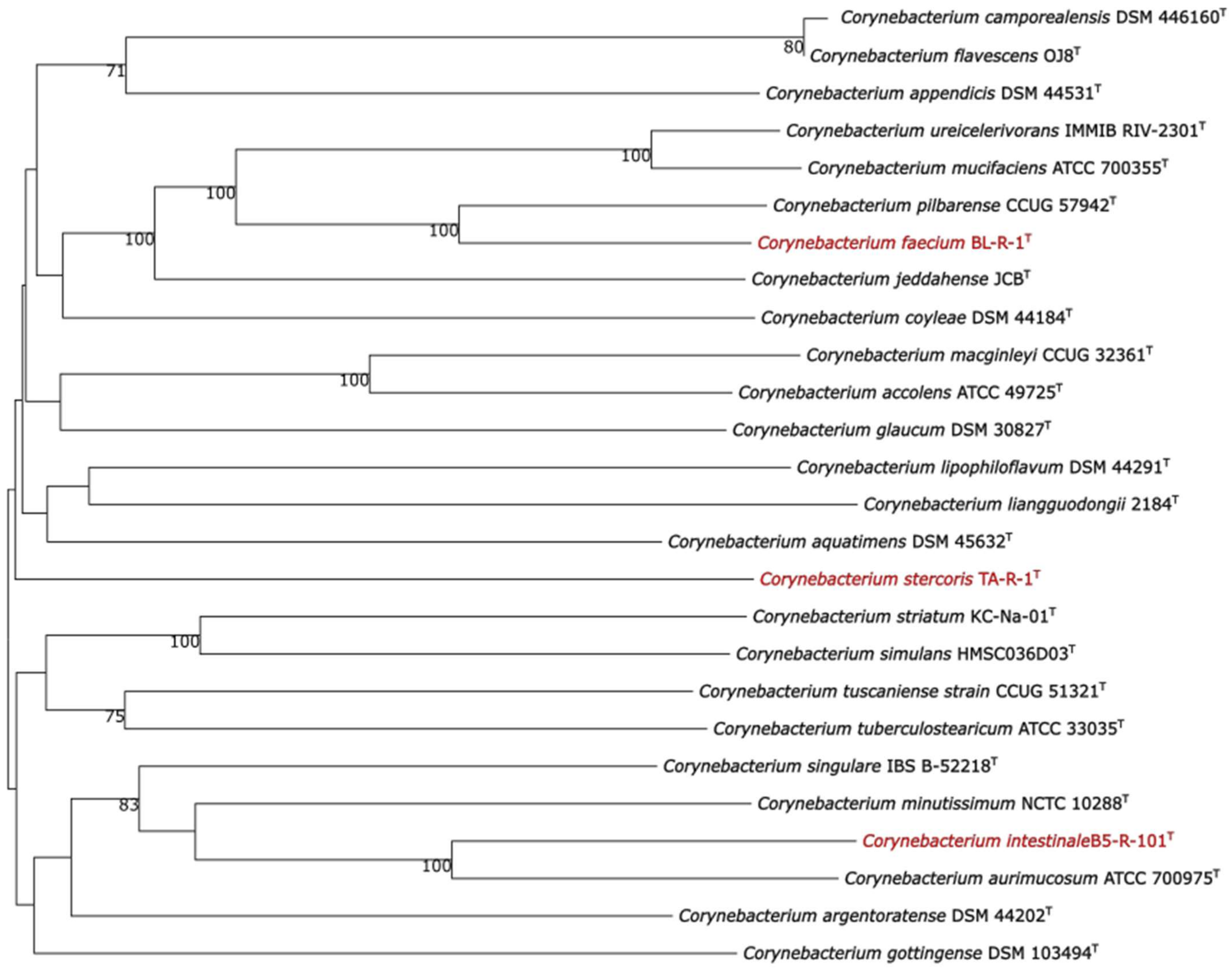

Supplementary Figure S7. Phylogenomic tree based on the whole genome sequence of strains B5-R-101^T, TA-R-1^T, and BL-R-1^T reconstructed using TYGS.

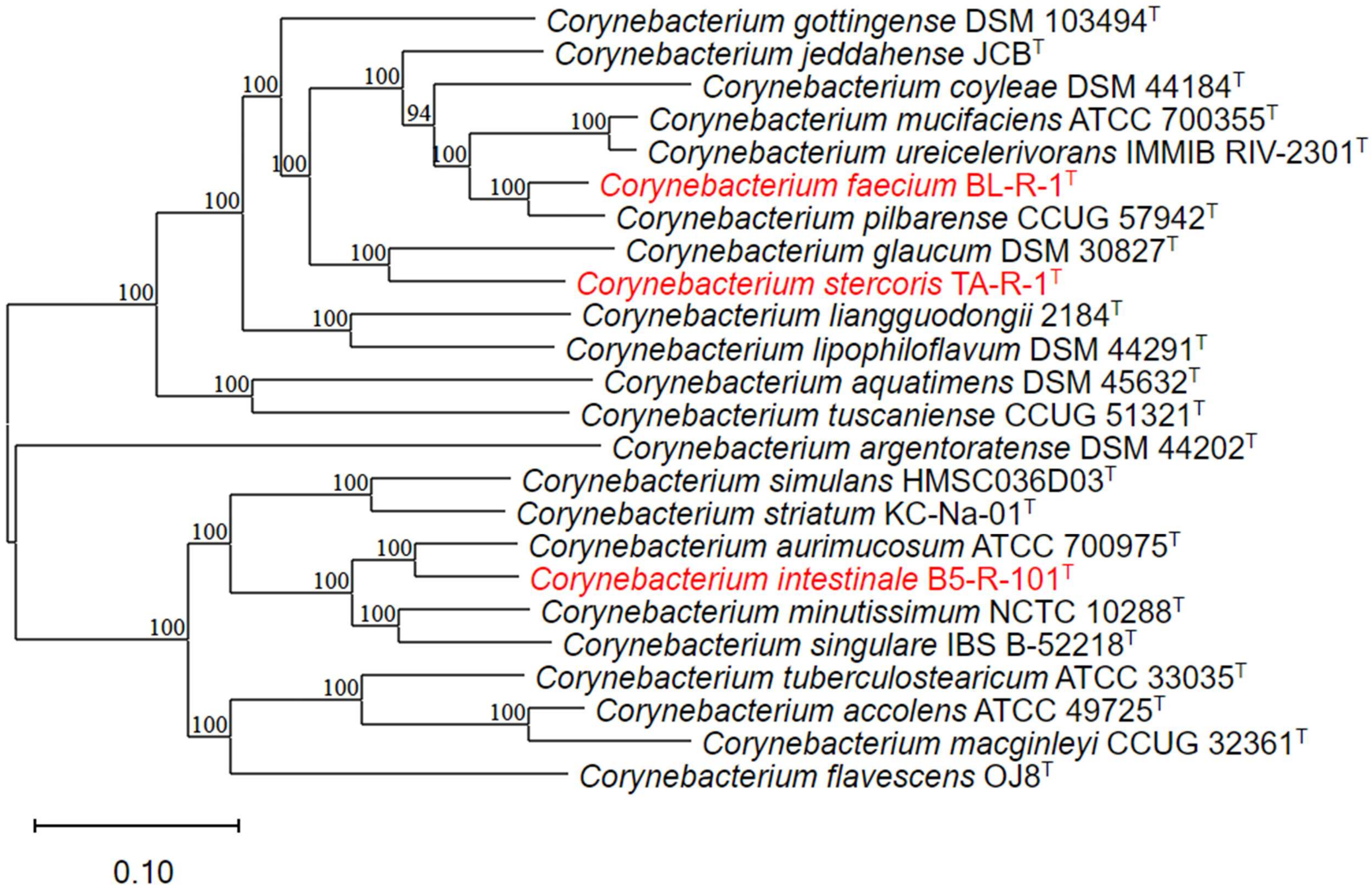

Supplementary Figure S8. Whole-genome-based phylogenetic tree were constructed using UBCGs and showing the phylogenomic relationship of strains B5-R-101^{T}, TA-R-1^{T}, and BL-R-1^{T} with closest validly published species of the genus *Corynebacterium.*

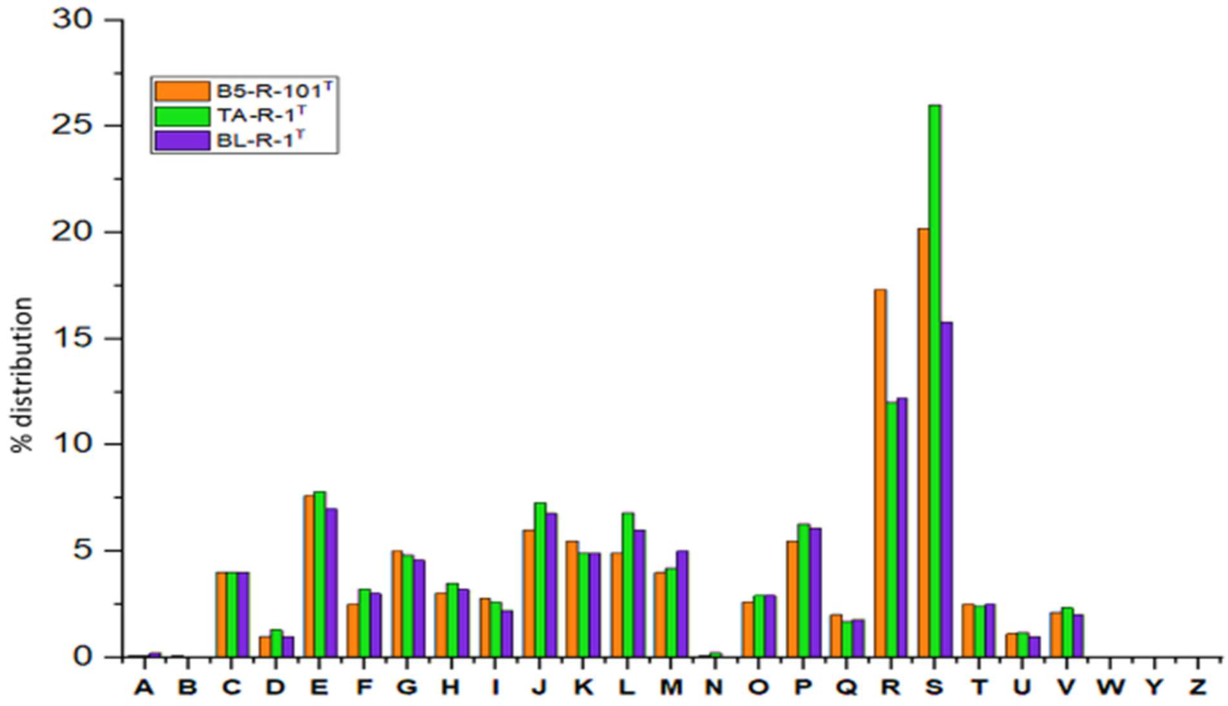

Supplementary Figures S9. Distribution of Cluster of Orthologous Group (COG) functional categories of the genome of strains B5-R-10^T, TA-R-1^T and BL-R-1^T, where A: RNA processing and modification. B: Chromatin structure and dynamics, C: Energy production and conversion, D: Cell cycle control, cell division, chromosome partitioning, E: Amino acid transport and metabolism, F: Nucleotide transport and metabolism, G: Carbohydrate transport and metabolism, H: Coenzyme transport and metabolism, Lipid transport and metabolism, J: Translation, ribosomal structure and biogenesis, K: Transcription, L: Replication, recombination and repair, M: Cell wall/membrane/envelope biogenesis, N: Cell mobility, O: Posttranslational modification, protein turnover, chaperones, P: Inorganic ion transport and metabolism, Q: Secondary metabolites biosynthesis transport and catabolism, R: General function prediction only, S: Function unknown, T: Signal transduction mechanisms, U: Intracellular trafficking, secretion, and vesicular transport, V: Defence mechanisms, W: Extracellular structures, Y: Nuclear structure, Z: Cytoskeleton

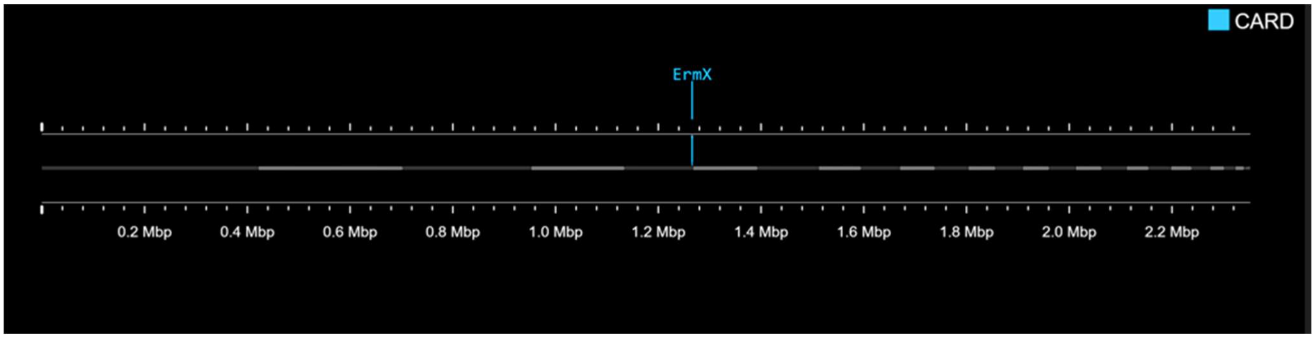

Supplementary Figure S10. Analysis of antibiotic resistance genes from strain TA-R-1^T by using CARD analysis.

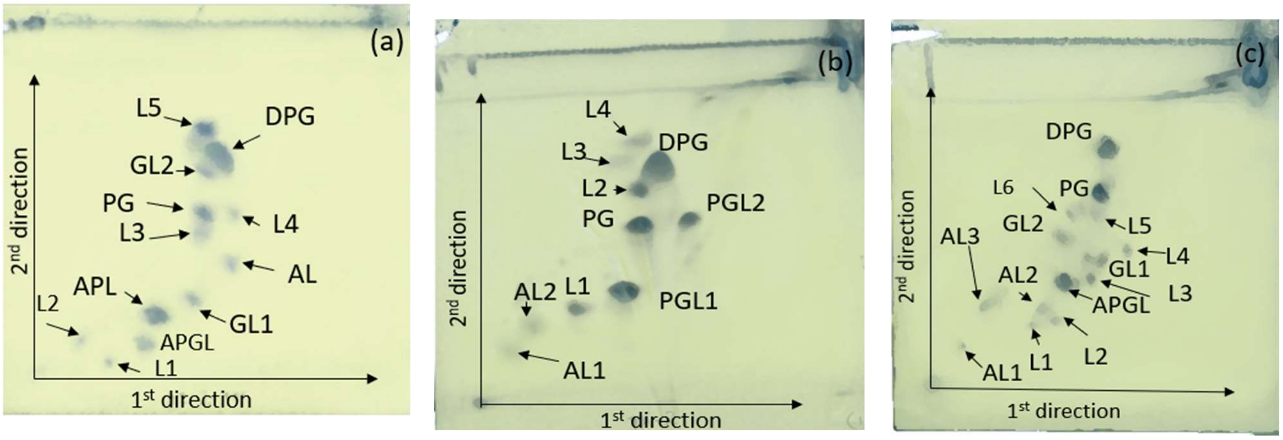

Supplementary Figure S11. Two-dimensional thin-layer chromatography of total polar lipids of strain (a) B5-R-101^T (b) TA-R-1^T and (c) BL-R-1^T. Chloroform-methanol-water (65:25:4) was used in the first direction, followed by chloroform-acetic acid-methanol-water (40:7.5:6:2) in the second direction. Abbreviations: DPG, diphosphatidylglycerol; PG, phosphatidylglycerol; PGL1-PGL2, unidentified phosphoglycolipids; GL1-GL2, unidentified glycolipids; AL1-AL3, unidentified amino lipids; APL, unidentified aminophospholipid; APGL, unidentified aminophosphoglycolipid and L1-L6, unidentified polar lipids.

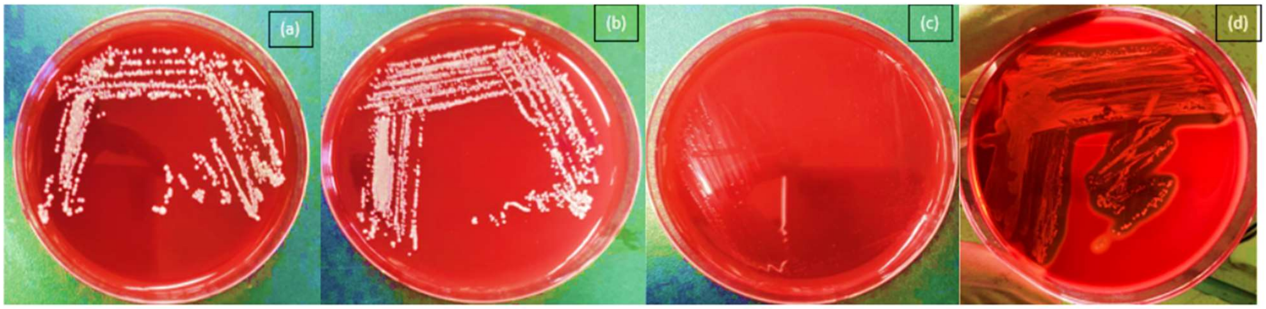

Supplementary Figure S12. Analysis of the hemolytic activity of strains B5-R-101^T (a), TA-R-1^T (b), BL-R-1^T (c), and *Cutibacterium acnes* KB112 (d). *C. acnes* KB112 was used as a positive control.

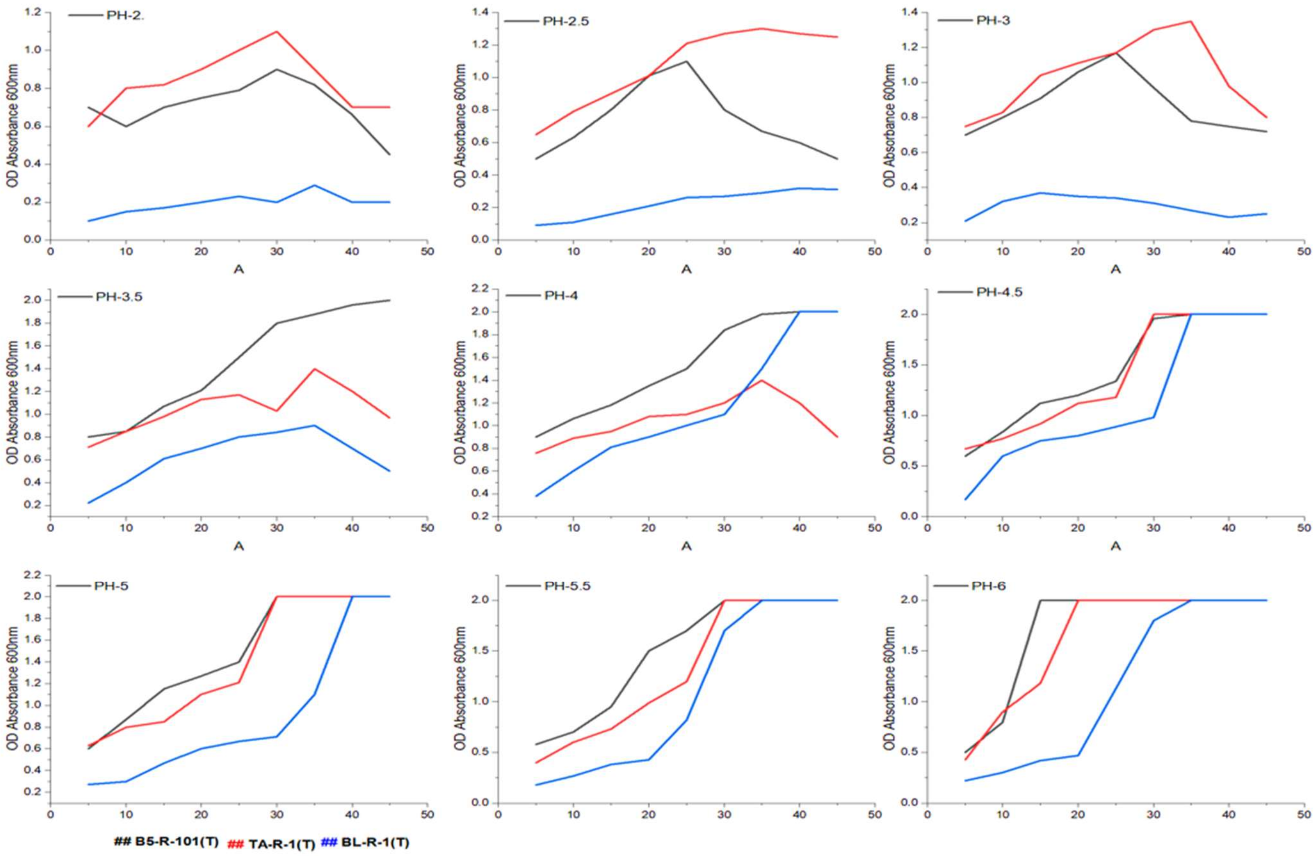

Supplementary Figure S13. Survival of the isolates under acidic pH conditions incubated in BHI media for 48 h at 37 °C.

Supplementary Table S1. DPPH + sample reaction mixture

The total reaction mixture (180 μL)	Sample(μL)	0.1mM DPPH (μL)	Solvent(μL)	MtoH, (μL)
Standard	-	90	90	-
Experiment	90	90	-	-
Control	90	-	-	90
Blank	-	-	90	90

Supplementary Table S2. 16S rRNA gene sequence similarities (%)

Strains	16S rRNA gene sequence similarities (%)	EzBioCloud	NCBI
**B5-R-101^T**	*Corynebacterium aurimucosum* NRRL B-24143^T	99.1	98.88
	Corynebacterium minutissimum ATCC 23348^T	98.82	98.5
	Corynebacterium singulare IBS B-52218^T	98.82	98.87
	Corynebacterium simulans UCL553^T	97.36	97.66
**TA-R-1^T**	*Corynebacterium aquatimens* IMMIB L-2475^T	98.73	98.33
	Corynebacterium tuscaniense ISS-5309 ^{T}	97.04	96.73
	'Corynebacterium bouchesdurhonense' SN14^T	97.88	96.49
	Corynebacterium gottingense DSM 103494^T	96.5	96.39
**BL-R-1^T**	*Corynebacterium mucifaciens* DMMZ 2278^T	98.32	97.79
	Corynebacterium pilbarense IMMIB WACC-658^T	98.46	98.22
	Corynebacterium ureicelerivorans IMMIB RIV-2301^T	97.99	97.66
	'Corynebacterium ihumii' GD7^T	97.78	97.46

Supplementary Table S3. List of genes in the genome of strains B5-R-101^T, TA-R-1^T and BL-R-1^T, which may have potential link for probiotics.

Gene	B5-R-101^T	TA-R-1^T	BL-R-1^T	Function of gene
luxS and *luxR*	fig|2943492.4.peg.969<br>fig|2943492.4.peg.1206	fig|2943490.3.peg.23	fig|6666666.978497.peg.847<br>fig|6666666.978497.peg.889	Quorum sensing, regulates gene expression for survival and beneficial molecule production (Ball et al., 2017).
clpB, clpX,clpS and *clpC*	fig|2943492.4.peg.1880	fig|2943490.3.peg.327<br>fig|2943490.3.peg.1257<br>fig|2943490.3.peg.324	fig|6666666.978497.peg.1680<br>fig|6666666.978497.peg.1683<br>fig|6666666.978497.peg.1746<br>fig|6666666.978497.peg.1804	Involved in stress response, survival, and probiotic activity of bacteriaby aiding protein degradation and homeostasis maintenance (Queraltó et al., 2023)
groEl groES dnaJ and *dnaG*	fig|2943492.4.peg.1925<br>fig|2943492.4.peg.2371	fig|2943490.3.peg.485<br>fig|2943490.3.peg.1520<br>fig|2943490.3.peg.1868<br>fig|2943490.3.peg.272<br>fig|2943490.3.peg.1662	fig|6666666.978497.peg.208<br>fig|6666666.978497.peg.2238<br>fig|6666666.978497.peg.209	Involved in protein folding, stress response, and quality control to ensure that probiotic bacteria produce stable and functional proteinsfor their beneficial effects on the host (Beck et al., 2009).
coPZ, copZA and *csoR*			fig|6666666.978497.peg.491<br>fig|6666666.978497.peg.1917	Manipulating copper homeostasis in probiotic bacteria through csoR regulation could lead to enhanced probiotic activity (Smaldone & Helmann, 2007).
Ferritin	fig|2943492.4.peg.1000	fig|2943490.3.peg.448	fig|6666666.978497.peg.726	Regulates its release, preventing excess iron from damaging cellular structures. Maintains gut iron homeostasis and oxidative stress tolerance(Arosio & Levi, 2002).
recA, recN, recR, recX and *RecN*	fig|2943492.4.peg.1323<br>fig|2943492.4.peg.1322<br>fig|2943492.4.peg.580<br>fig|2943492.4.peg.1108	fig|2943490.3.peg.443	fig|6666666.978497.peg.1047<br>fig|6666666.978497.peg.1303	DNA repair and genome stability (Herbel et al., 2013)
mutT, muTT and *mutT3*	fig|2943492.4.peg.310<br>fig|2943492.4.peg.401<br>fig|2943492.4.peg.243<br>fig|2943492.4.peg.1905	fig|2943490.3.peg.1694<br>fig|2943490.3.peg.194		Prevent replication errors for genome stability (Da Silva et al., 2019).
Yidc		fig|2943490.3.peg.738<br>fig|52943490.3.peg.1550	fig|6666666.978497.peg.603<br>fig|6666666.978497.peg.604	Involved in protein folding, membrane integrity, cell signalling, molecule transport, energy metabolism, and potentially antibiotic resistance (Stergiou et al., 2021)
sitB		fig|2943490.3.peg.1285		involved in the production and secretion of bacteriocins.
rmuC	fig|2943492.4.peg.169			DNA repair (Da Silva et al., 2019)

atpA, atpB, atpD, and *atpF*	fig|2943492.4.peg.297	fig|2943490.3.peg.338	fig|6666666.978497.peg.360	F0F1-type ATP synthase(Curtis, 1988)
hrcA	fig|2943492.4.peg.1707	fig|2943490.3.peg.273	fig|6666666.978497.peg.1612	HrcA is a heat shock protein found in some probiotic bacteria like Lactobacillus acidophilus that protects the bacteria from environmental stressors (Bucka-Kolendo et al., 2021).
lytR, cpsA and *psr*	fig|2943492.4.peg.942<br>fig|2943492.4.peg.1158	fig|2943490.3.peg.157<br>fig|2943490.3.peg.204	fig|6666666.978497.peg.107<br>fig|6666666.978497.peg.1914	lytR, cpsA, and psr are proteins found in some probiotic bacteria that play important roles in maintaining structural integrity, protection against environmental stressors, and adhesion to host tissues (D'Abrosca et al., 2018).
lspA- lipoprotein	fig|2943492.4.peg.1474	fig|2943490.3.peg.120<br>fig|2943490.3.peg.1524<br>fig|2943490.3.peg.380	fig|6666666.978497.peg.1409<br>fig|6666666.978497.peg.1737	Lipoproteins in probiotic bacteria play important roles in maintaining cell wall integrity, adhesion to host tissues, and modulating the host immune response (Tsuji et al., 2023).
Translation elongation factor (lePA, TU,Ts,G,P)	fig|2943492.4.peg.1386	fig|2943490.3.peg.44<br>fig|2943490.3.peg.299<br>fig|2943490.3.peg.1061<br>fig|2943490.3.peg.1474<br>fig|2943490.3.peg.1944<br>fig|2943490.3.peg.1945	fig|6666666.978497.peg.290<br>fig|6666666.978497.peg.291<br>fig|6666666.978497.peg.1143<br>fig|6666666.978497.peg.1482<br>fig|6666666.978497.peg.1640	Translation elongation factor (EF-Tu) is a protein found in some probiotic bacteria that plays a role in protein synthesis, and also interacts with host cells and contributes to the modulation of the host immune response (Harvey et al., 2019).
Sortase A, LPXTG specific	fig|2943492.4.peg.1837<br>fig|2943492.4.peg.1834		fig|6666666.978497.peg.2228<br>fig|6666666.978497.peg.2231	Sortase A is an enzyme found in some probiotic bacteria that anchors cell surface proteins, contributing to cell wall integrity, adhesion to host tissues, and modulation of the host immune response (Susmitha et al., 2021).
gpr	fig|2943492.4.peg.167<br>fig|2943492.4.peg.848<br>fig|2943492.4.peg.1657	fig|2943490.3.peg.310<br>fig|2943490.3.peg.823	fig|6666666.978497.peg.865<br>fig|6666666.978497.peg.1656<br>fig|6666666.978497.peg.1973	Encoding G-protein coupled receptors found in some probiotic bacteria, interacts with host cells to modulate the immune response, and contribute to the beneficial effects of probiotics.
cfa	fig|2943492.4.peg.538<br>fig|2943492.4.peg.2408	fig|2943490.3.peg.929<br>fig|2943490.3.peg.1619	fig|6666666.978497.peg.1875	Encodes for cyclopropane-fatty-acyl-phospholipid that enhances lipid synthesis (Kandasamy et al., 2022).
Inorganic pyrophosphatase	fig|2943492.4.peg.1943	fig|2943490.3.peg.50	fig|6666666.978497.peg.2212	Inorganic pyrophosphatase that maintains surface tension (Kandasamy et al., 2022).
glpX	fig|2943492.4.peg.162	fig|2943490.3.peg.1820	fig|6666666.978497.peg.869	promotes energy and gut health in probiotics (M. He & Shi, 2017).

Supplementary Table S4. List of genes in the genome of strains B5-R-101^T, TA-R-1^T and BL-R-1^T that may have antioxidant characteristics.

Antioxidant	B5-R-101^T	TA-R-1^T	BL-R-1^T	Functions
katE	fig|2943492.4.peg.1121	fig|2943490.3.peg.430	fig|6666666.978497.peg.118<br>fig|6666666.978497.peg.640	Breaks down hydrogen peroxide into water and oxygen, helping protect against oxidative damage. (Borisov et al., 2021)
ahpD		fig|2943490.3.peg.1139	fig|6666666.978497.peg.191<br>fig|6666666.978497.peg.1072	Neutralizing harmful ROS and converting them into less toxic compounds.(Hillas et al., 2000)
orn	fig|2943492.4.peg.1593		fig|6666666.978497.peg.1717	Produces oxireductase enzyme that helps bacteria cope with oxidative stress and adapt to different environments(Petrov et al., 2010).
glutathione (cydA and *yocS)*		fig|2943490.3.peg.95		protecting cells from oxidative stress by acting as an antioxidant(Teramoto et al., 2013).
feoB and *oxyR*	fig|2943492.4.peg.2340<br>fig|2943492.4.peg.688	fig|2943490.3.peg.2145<br>fig|2943490.3.peg.1141	fig|6666666.978497.peg.1691<br>fig|6666666.978497.peg.1070	Regulates iron levels and prevents formation of ROS, low-pH-induced(J. He et al., 2006)
katE	fig|2943492.4.peg.1121			Breaks down hydrogen peroxide and protects against oxidative damage.
sufC, sufD, sufR and *sufE2*			fig|2943490.3.peg.1004<br>fig|2943490.3.peg.1003<br>fig|2943490.3.peg.1002<br>fig|2943490.3.peg.999	Iron-sulfur cluster assembly, *ahpC, ahpD,* and *ahpF* work together to detoxify ROS and other harmful compounds(Saini et al., 2010).
glO1	fig|2943490.3.peg.1079			Glutathione: Non-redox reactions, Methylglyoxal Metabolism (Distler & Palmer, 2012)
Thioredoxin	fig|2943492.4.peg.304<br>fig|2943492.4.peg.2162<br>fig|2943492.4.peg.2050<br>fig|2943492.4.peg.1043	fig|2943492.4.peg.755<br>fig|2943490.3.peg.730<br>fig|2943490.3.peg.731<br>ig|2943490.3.peg.773<br>fig|2943490.3.peg.1651<br>fig|2943490.3.peg.1358	fig|6666666.978497.peg.489<br>fig|6666666.978497.peg.598<br>fig|6666666.978497.peg.415<br>fig|6666666.978497.peg.2086	Thioredoxin is a key antioxidant system in defence against oxidative stress through its disulfide reductase activity regulating protein dithiol/disulfide balance(Liu et al., 2020).
mntR, sitD, sitA and *sitB*	fig|2943492.4.peg.1256<br>fig|2943492.4.peg.1630	fig|2943490.3.peg.1296	fig|6666666.978497.peg.323<br>fig|6666666.978497.peg.321<br>fig|6666666.978497.peg.322<br>fig|6666666.978497.peg.30	Protecting bacteria from oxidative stress by regulating the expression of genes involved in synthesising and repairing iron-sulfur clusters are important cofactors in antioxidant enzymes(Liu et al., 2020).
NADH	fig|2943492.4.peg.539<br>fig|2943492.4.peg.1319<br>fig|2943492.4.peg.2317	fig|2943490.3.peg.69<br>fig|2943490.3.peg.608<br>fig|2943490.3.peg.928<br>fig|2943490.3.peg.2008	fig|6666666.978497.peg.69	NADH is a co enzyme that helps produce energy, and methionine sulfoxide reductase genes encode enzymes that repair damaged proteins caused by oxidative stress (Spaans et al., 2015)
nrdH, nrdR, and *nrdI*	fig|2943492.4.peg.1543	fig|2943490.3.peg.400	fig|6666666.978497.peg.1756<br>fig|6666666.978497.peg.1068<br>fig|6666666.978497.peg.1755	The *nrdH, nrdR,* and *nrdI* genes play a role in antioxidant defense in bacteria by regulating the expression of genes involved in the synthesis of ribonucleotide reductase(Torrents, 2014)

pyruvate oxidase	fig|2943492.4.peg.590<br>fig|2943492.4.peg.591<br>fig|2943492.4.peg.1057<br>fig|2943492.4.peg.1568	fig|2943490.3.peg.1789<br>fig|2943490.3.peg.219<br>fig|2943490.3.peg.87<br>fig|2943490.3.peg.176	fig|6666666.978497.peg.2126<br>fig|6666666.978497.peg.133<br>fig|6666666.978497.peg.1268<br>fig|6666666.978497.peg.1267	Pyruvate oxidase in bacteria plays a role in antioxidant defense by generating hydrogen peroxide, which can induce expression of antioxidant genes and protect the bacterial cell from oxidative stress(Bruno-Bárcena et al., 2010).
msrA and *msrB*	fig|2943492.4.peg.722<br>fig|2943492.4.peg.1771	fig|2943490.3.peg.1107<br>fig|2943490.3.peg.1107	fig|6666666.978497.peg.1944	The main function of *msrA* and *msrB* is to protect bacterial cells from oxidative stress by reducing and repairing oxidized proteins, making them crucial for the cell's antioxidant defense system

Supplementary Table S5. Predicted biosynthetic gene cluster for secondary metabolites in strains B5-R-101^T, TA-R-1^T, and BL-R-1^T.

Isolated strain	Reference	Type	Compound(s)	Organism
B5-R-101^T<br>NZ_JAMFTR010000004.1<br>BGC0000648: 1057-11443<br>Rejoin -4.1<br>Location: 1 - 16,097 nt.<br>(total: 16,097 nt)	BGC0000648)<br>BGC0000650<br>BGC0000637<br>BGC0000640<br>BGC0000644<br>BGC0000635<br>BGC0000630<br>BGC0000664<br>BGC0002656	Terpene<br>Polyketide	carotenoid<br>(2R,3S,3'S)-2-hydroxyastaxanthin<br>isorenieratene<br>oryzanaphthopyran A, oryzanaphthopyran B, oryzanaphthopyran C, oryzanthrone A, oryzanthrone B, chlororyzanthrone A, chlororyzanthrone B	*Myxococcus xanthus*<br>*Algoriphagus* sp. KK10202C<br>*Corynebacterium glutamicum*<br>*Enterobacteriaceae bacterium* DC404<br>*Dietzia* sp. CQ4<br>*Paracoccus* sp. N81106<br>*Paracoccus haeundaensis*<br>*Streptomyces griseus* subsp. griseus NBRC 13350<br>*Streptacidiphilus oryzae* TH49
B5-R-101^T<br>NZ_JAMFTR010000005 - Region 1 - T1PKS<br>Location: 41,005 - 85,777 nt. (total: 44,773 nt)	BGC0000513<br>BGC0002405<br>BGC0001410<br>BGC0001909<br>BGC0001831<br>BGC0001163<br>BGC0001162<br>BGC0001161<br>BGC0000044<br>BGC0002547	RiPP<br>Saccharide<br>Polyketide	Ery-9, Ery-6, Ery-8, Ery-7, Ery-5, Ery-4, Ery-3<br>mid-chain acyl sugars<br>polysaccharide A<br>strobilurin A<br>alkylpyrone-407, alkylpyrone-393<br>1-heptadecene<br>1-heptadecene<br>1-heptadecene<br>dawenol<br>youssoufene A1, youssoufene B1, youssoufene B2, youssoufene B3, youssoufene B4	*Saccharopolyspora erythraea* NRRL 2338<br>*Solanum lycopersicum*<br>*Bacteroides fragilis*<br>*Strobilurus tenacellus*<br>*Myxococcus xanthus* DK 1622<br>*Moorea producens* 3L<br>*Moorea producens* JHB<br>*Moorea bouillonii* PNG<br>*Stigmatella aurantiaca* DW4/3-1<br>*Streptomyces youssoufiensis*
B5-R-101^T<br>NZ_JAMFTR010000006 - Region 1 – NAPAA<br>Location: 30,333 - 64,295 nt. (total: 33,963 nt)	BGC0001641<br>BGC0001844<br>BGC0001132<br>BGC0001641<br>BGC0002536<br>BGC0002174<br>BGC0002535<br>BGC0000343<br>BGC0002476<br>BGC0000375<br>BGC0002295<br>BGC0000131	NRP,<br>Saccharide<br>Polyketide	kolossin<br>holrhizin<br>xenotetrapeptide<br>kolossin<br>alazopeptin<br>ε-Poly-L-lysine<br>γ-poly-L-2,4-diaminobutyric acid<br>enterobactin<br>enterobactin<br>indigoidine<br>minimycin<br>pyrrolomycin	*Photorhabdus laumondii* subsp. laumondii TTO1<br>*Paraburkholderia rhizoxinica* HKI 454<br>*Xenorhabdus nematophila* ATCC 19061<br>*Photorhabdus laumondii* subsp. laumondii TTO1<br>*Kitasatospora azatica* KCTC 9699<br>*Epichloe festucae*<br>*Streptomyces celluloflavus*<br>*Pseudomonas* sp. J465<br>*Escherichia coli* str. K-12 substr. MG1655<br>*Streptomyces chromofuscus*<br>*Streptomyces hygroscopicus*<br>*Streptomyces* sp. UC 11065

TA-R-1 T<br>NZ_JAMFTQ010000004 - Region 1 - T1PKS Location: 53,279 - 98,045 nt. (total: 44,767 nt)	BGC0000892<br>BGC0002476<br>BGC0001909<br>BGC0001831<br>BGC0001897<br>BGC0000056<br>BGC0002477<br>BGC0000410<br>BGC0002547	Other<br>NRP<br>Polyketide	caryoynencin<br>enterobactin<br>strobilurin A<br>alkylpyrone-407, alkylpyrone-393<br>cepacin A<br>esperamicin<br>thioangucycline A, thioangucycline B<br>pseudomonine<br>youssoufene A1, youssoufene B1, youssoufene B2, youssoufene B3, youssoufene B4	*Burkholderia gladioli BSR3*<br>*Escherichia coli str.* K-12 substr. MG1655<br>*Strobilurus tenacellus*<br>*Myxococcus xanthus* DK 1622<br>*Burkholderia ambifaria* IOP40-10<br>*Actinomadura verrucosospora*<br>*Streptomyces* sp. CB00072<br>*Pseudomonas fluorescens*<br>*Streptomyces youssoufiensis*
TA-R-1T<br>NZ_JAMFTQ010000005 - Region 1 – terpene Location: 95,899 - 116,711 nt. (total: 20,813 nt)	BGC0001483<br>BGC0000648<br>BGC0000650<br>BGC0000647<br>BGC0000664<br>BGC0000633<br>BGC0000637<br>BGC0000646<br>BGC0000640<br>BGC0000630<br>BGC0001456	Terpene,	5-isoprenylindole-3-carboxylate β-D-glycosyl ester<br>carotenoid<br>carotenoid<br>carotenoid<br>isorenieratene<br>carotenoid<br>carotenoid<br>β-carotein<br>carotenoid<br>(2R,3S,3'S)-2-hydroxyastaxanthin<br>isorenieratene	*Streptomyces* sp. RM-5-8<br>*Myxococcus xanthus*<br>*Algoriphagus* sp. KK10202C<br>*Rhodobacter sphaeroides*<br>*Streptomyces griseus* subsp. griseus NBRC 13350<br>*Streptomyces avermitilis*<br>*Corynebacterium glutamicum*<br>*uncultured bacterium*<br>*Enterobacteriaceae bacterium* DC404<br>*Paracoccus haeundaensis*<br>*Streptomyces argillaceus*
TA-R-1T<br>NZ_JAMFTQ010000006 - Region 1 - RiPP-like Location: 1 - 8,701 nt. (total: 8,701 nt)	BGC0002150<br>BGC0002151<br>BGC0000504<br>BGC0000554<br>BGC0002322<br>BGC0001743<br>BGC0002306<br>BGC0001222<br>BGC0000491<br>BGC0002488	RiPP	triculamin<br>alboverticillin<br>cytolysin ClyLl, cytolysin ClyLs<br>SRO15-3108<br>citrocin<br>plantaricyclin<br>freyrasin<br>acidocin B<br>gassericin A<br>bacillopaline	*Streptomyces triculaminicus*<br>*Streptomyces griseocarneus*<br>Plasmid pAD1<br>*Streptomyces filamentosus* NRRL 15998<br>*Citrobacter pasteurii*<br>*Lactobacillus plantarum*<br>*Paenibacillus polymyxa* ATCC 842<br>*Lactobacillus acidophilus*<br>*Lactobacillus gasseri*<br>*Paenibacillus mucilaginosus* KNP414
TA-R-1T<br>NZ_JAMFTQ010000011 - Region 1 – NAPAA Location: 10,782 - 44,756 nt. (total: 33,975 nt)	BGC0002174<br>BGC0002535<br>BGC0001137<br>BGC0002476<br>BGC0000375<br>BGC0002295<br>BGC0000925<br>BGC0000410<br>BGC0000131<br>BGC0002694	NRP<br>Alkaloid<br>Saccharide<br>Polyke	ε-Poly-L-lysine<br>γ-poly-L-2,4-diaminobutyric acid<br>marinacarboline A, marinacarboline B, marinacarboline C, marinacarboline D<br>enterobactin<br>indigoidine<br>minimycin<br>quinolobactin<br>pseudomonine<br>pyrrolomycin<br>roseobactin	*Epichloe festucae*<br>*Streptomyces celluloflavus*<br>*Marinactinospora thermotolerans*<br>*Escherichia coli str. K-12 substr. MG1655*<br>*Streptomyces chromofuscus*<br>*Streptomyces hygroscopicus*<br>*Pseudomonas fluorescens*<br>*Pseudomonas fluorescens*<br>*Streptomyces* sp. UC 11065<br>*Phaeobacter inhibens* 2.10

BL-R-1^T<br>NZ_JAPYJX010000001 - Region 1 – ectoine<br>Location: 16,186 - 26,560 nt. (total: 10,375 nt)	BGC0000855<br>BGC0000859<br>BGC0000917<br>BGC0000857<br>BGC0000854<br>BGC0001778<br>BGC0000925<br>BGC0002080<br>BGC0002097<br>BGC0001073	(Amino acid-derived)<br>NRP, Polyketide, Saccharide<br>NRP, Polyketide<br>Type	ectoine<br>ectoine<br>molybdenum cofactor<br>ectoine<br>ectoine<br>showdomycin<br>quinolobactin<br>stieleriacine A1, stieleriacine A2, stieleriacine B1, stieleriacine B2, stieleriacine C<br>kosinostatin<br>kosinostatin<br>Compound(s)	*Methylomicrobium kenyense*<br>*Methylomicrobium alcaliphilum*<br>*Rhodobacter capsulatus*<br>*Methylophaga alcalica*<br>*Methylomicrobium alcaliphilum 20Z*<br>*Streptomyces showdoensis*<br>*Pseudomonas fluorescens*<br>*Stieleria maiorica*<br>*Micromonospora* sp. 28ISP2-46<br>*Micromonospora* sp. TP-A04
BL-R-1^T<br>NZ_JAPYJX010000002 - Region 1 – terpene<br>Location: 149,685 - 170,494 nt. (total: 20,810 nt)	BGC0000650<br>BGC0000648<br>BGC0000637<br>BGC0000640<br>BGC0000630<br>BGC0000664<br>BGC0000633<br>BGC0000644<br>BGC0001227<br>BGC0000645	Terpene	carotenoid<br>carotenoid<br>carotenoid<br>carotenoid<br>(2R,3S,3'S)-2-hydroxyastaxanthin<br>isorenieratene<br>carotenoid<br>carotenoid<br>isorenieratene<br>carotenoid	*Algoriphagus* sp. KK10202C<br>*Myxococcus xanthus*<br>*Corynebacterium glutamicum*<br>*Enterobacteriaceae bacterium DC404*<br>*Paracoccus haeundaensis*<br>*Streptomyces griseus* subsp. griseus NBRC 13350<br>*Streptomyces avermitilis*<br>*Dietzia* sp. CQ4<br>*Streptomyces collinus* Tu 365<br>*Halobacillus halophilus* DSM 2266
BL-R-1^T<br>NZ_JAPYJX010000007 - Region 1 - T1PKS<br>Location: 101,448 - 146,157 nt. (total: 44,710 nt)	BGC0000892<br>BGC0001897<br>BGC0001909<br>BGC0002547<br>BGC0000056<br>BGC0001161<br>BGC0001163<br>BGC0001162<br>BGC0002476<br>BGC0002016	NRP<br>Polyketide	caryoynencin<br>cepacin A<br>strobilurin A<br>youssoufene A1, youssoufene B1, youssoufene B2, youssoufene B3, youssoufene B4<br>esperamicin<br>1-heptadecene<br>1-heptadecene<br>1-heptadecene<br>enterobactin<br>lugdunomycin	*Burkholderia gladioli* BSR3<br>*Burkholderia ambifaria* IOP40-10<br>*Strobilurus tenacellus*<br>*Streptomyces youssoufiensis*<br>*Actinomadura verrucosospora*<br>*Moorea bouillonii* PNG<br>*Moorea producens* 3L<br>*Moorea producens* JHB<br>*Escherichia coli* str. K-12 substr. MG1655<br>*Streptomyces* sp. QL37
BL-R-1^T<br>NZ_JAPYJX010000009 - Region 1 – NAPAA<br>Location: 85,238 - 119,032 nt. (total: 33,795 nt)	BGC0002174<br>BGC0002535<br>BGC0001758<br>BGC0001844<br>BGC0001128<br>BGC0002518<br>BGC0001641<br>BGC0001132<br>BGC0002437<br>BGC0002286	NRP	ε-Poly-L-lysine<br>γ-poly-L-2,4-diaminobutyric acid<br>rhizomide A, rhizomide B, rhizomide C<br>holrhizin<br>gamexpeptide C<br>syringafactin A, syringafactin C<br>kolossin<br>xenotetrapeptide<br>thermoactinoamide A	*Epichloe festucae*<br>*Streptomyces celluloflavus*<br>*Paraburkholderia rhizoxinica* HKI 454<br>*Paraburkholderia rhizoxinica* HKI 454<br>*Photorhabdus laumondii* subsp. laumondii TTO1<br>*Pseudomonas* sp. SZ57<br>*Photorhabdus laumondii* subsp. laumondii TTO1<br>*Xenorhabdus nematophila* ATCC 19061<br>*Thermoactinomyces* sp. AS95

			ririwpeptide A, ririwpeptide B, ririwpeptide C	*Photorhabdus laumondii* subsp. laumondii TTO1

Supplementary Table S6. Cellular fatty acid profiles (% of the total) of the novel strains of the genus *Corynebacterium* and related type strains. The strains included in the table are as follows: 1, B5-R-101^T; 2, TA-R-1^T; 3, BL-R-1^T; 4, *C. aurimucosum* DSM 44532^T, 5. *C. minutissimum* DSM 20651^T; 6, *C. singulare* DSM 44357^T; 7, *C. aquatimens* DSM 45632^T; 8, *C. ureicelerivorans* DSM 45051^T; and 9, *C. mucifaciens* DSM 44265^T. All data were obtained from this study. TR, trace amount (<1%); –, not detected.

Fatty acids	1	2	3	4	5	6	7	8	9
**Saturated**									
$C_{14:0}$	–	TR	TR	2.3	1.2	TR	1.9	1.0	TR
$C_{16:0}$	14.1	29.7	30.6	43.3	32.7	21.8	29.0	35.6	38.6
$C_{17:0}$	–	2.4	3.1	TR	1.9	1.8	TR	TR	1.6
$C_{18:0}$	–	5.7	8.9	2.5	3.6	3.0	5.8	5.1	4.1
**Unsaturated**									
$C_{13:1}$ at 12-13	6.3	–	–	–	–	–	–	–	–
$C_{15:1}$ $\omega 6c$	3.7	TR	TR	–	TR	TR	–	–	TR
$C_{16:1}$ $\omega 9c$	–	1.4	TR	TR	2.1	1.1	2.5	–	3.5
$C_{17:1}$ $\omega 6c$	–	1.0	TR	TR	1.0	2.0	TR	–	TR
$C_{18:1}$ $\omega 7c$ 11-methyl	2.1	–	–	–	–	–	–	–	–
$C_{18:1}$ $\omega 9c$	18.8	28.7	36.8	21.2	24.6	19.5	38.3	48.4	21.1
$C_{20:1}$ $\omega 9c$	4.1	TR	TR	TR	TR	TR	TR	–	–
$C_{20:4}$ $\omega 6,9,12,15c$	–	TR	1.0	TR	TR	1.2	TR	1.0	TR
**Branched saturated**									
iso-$C_{14:0}$	–	–	TR	6.6	–	TR	TR	TR	TR
iso-$C_{15:0}$	–	–	TR	6.7	–	–	–	TR	TR
iso-$C_{16:0}$	–	–	TR	2.8	–	–	–	TR	–
iso-$C_{17:0}$	5.5	–	TR	TR	TR	1.0	TR	TR	TR
iso-$C_{19:0}$	–	2.5	–	TR	1.9	2.9	1.1	–	1.5
iso-$C_{20:0}$	1.9	–	–	TR	–	–	–	–	–
anteiso-$C_{11:0}$	6.1	TR	TR	TR	TR	TR	TR	TR	TR
anteiso-$C_{13:0}$	–	–	–	1.5	TR	–	–	–	TR
anteiso-$C_{14:0}$	2.4	–	–	TR	–	–	–	–	TR
anteiso-$C_{19:0}$	–	12.2	–	TR	15.5	21.4	13.3	–	1.6
anteiso-$C_{16:0}$	1.0	–	–	–	–	TR	–	–	tr
anteiso-$C_{17:0}$	1.6	1.0	1.7	TR	TR	2.7	TR	–	4.4
$C_{18:0}$ 10-methyl, TBSA	–	7.8	5.9	6.2	6.8	8.4	3.3	5.2	9.3
**Hydroxy**									

iso-$C_{11:0}$ 3-OH		1.3	–	–	–	–	–	–	–	–
iso-$C_{15:0}$ 3-OH		6.4	–	TR	TR	TR	–	–	–	–
iso-$C_{16:0}$ 3-OH		3.2	–	–	TR	TR	TR	1.0	–	TR
iso-$C_{17:0}$ 3-OH		3.7	–	–	–	–	4.5	–	–	–
**Branched unsaturated**										
iso-$C_{15:1}$ $\omega 9c$		4.1	–	–	–	–	–	–	–	–
iso-$C_{19:1}$ I		–	–	TR	–	TR	–	TR	TR	1.1
**Summed Features***										
	3	4.2	TR	2.7	TR	TR	TR	TR	–	–
	5	TR	–	–	–	–	tr	–	–	–
	7	4.9	1.0	1.1	TR	1.1	1.7	TR	TR	4.0
	9	2.8	–	–	–	–	–	TR	–	1.9

*Summed features represent groups of two or three fatty acids that could not be separated using the MIDI system. Summed feature 3 comprised $C_{16:1}\omega 7c$ and/or $C_{16:1}\omega 6c$, summed feature 5 comprised anteiso-$C_{18:0}$ and/or $C_{18:2}\omega 6,9c$, summed feature 7 comprised $C_{19:1}\omega 7c$ and/or $C_{19:1}\omega 6c$, and summed feature 9 comprised iso-$C_{17:1}$ $\omega 9c$ and/or $C_{16:0}$10-methyl.

Supplementary Table S7. ANI and dDDH (%) values of strains B5-R-101^T, TA-R-1^T, and BL-R-1^T against closest members of the genus *Corynebacterium*.

Strains	Closest members	ANI (%)	dDDH (%)
**B5-R-101^T**	*Corynebacterium aurimucosum* IMMIB D-1488^T	87.96	33.7
	Corynebacterium minutissimum NCTC 10288^T	82.71	25.1
	Corynebacterium singulare IBS B-52218^T	81.93	25
**TA-R-1$^{T.}$**	*Corynebacterium aquatimens* IMMIBL-2475^T	73.98	23
	Corynebacterium bouchesdurhonense SN14^T	79.24	22.8
	Corynebacterium tuscaniense ISS-5309 ^{T}	71.77	19.9
**BL-R-1^T**	*Corynebacterium haemomassiliense* Marseille-Q3615^T	91.24	63.4
	Corynebacterium pilbarense IMMIB WACC-658^T	71.77	43
	Corynebacterium mucifaciens DMMZ 2278^T	84.6	27.6

References:

Arosio, P., & Levi, S. (2002). Ferritin, iron homeostasis, and oxidative damage, *Free Radical Biology and Medicine*, *33*(4), 457–463. https://doi.org/10.1016/S0891-5849(02)00842-0

Ball, A. S., Chaparian, R. R., & van Kessel, J. C. (2017). Quorum sensing gene regulation by LuxR/HapR master regulators in vibrios. *Journal of Bacteriology*, *199*(19). https://doi.org/10.1128/JB.00105-17/ASSET/217225EB-D7B9-49BA-AB62-0A18EEC22CDA/ASSETS/GRAPHIC/ZJB9990944610003.JPEG

Beck, H. C., Madsen, S. M., Glenting, J., Petersen, J., Israelsen, H., Nørrelykke, M. R., Antonsson, M., & Hansen, A. M. (2009). Proteomic analysis of cell surface-associated proteins from probiotic *Lactobacillus plantarum*. *FEMS Microbiology Letters*, *297*(1), 61–66. https://doi.org/10.1111/J.1574-6968.2009.01662.X

Borisov, V. B., Siletsky, S. A., Nastasi, M. R., & Forte, E. (2021). ROS Defense Systems and Terminal Oxidases in Bacteria. *Antioxidants 2021, Vol. 10, Page 839*, *10*(6), 839. https://doi.org/10.3390/ANTIOX10060839

Bruno-Bárcena, J. M., Andrea Azcárate-Peril, M., & Hassan, H. M. (2010). Role of antioxidant enzymes in bacterial resistance to organic acids. *Applied and Environmental Microbiology*, *76*(9), 2747–2753. https://doi.org/10.1128/AEM.02718-09

Bucka-Kolendo, J., Juszczuk-Kubiak, E., & Sokołowska, B. (2021). Effect of High Hydrostatic Pressure on Stress-Related dnaK, hrcA, and ctsR Expression Patterns in Selected Lactobacilli Strains. *Genes 2021, Vol. 12, Page 1720*, *12*(11), 1720. https://doi.org/10.3390/GENES12111720

Curtis, S. E. (1988). Structure, organization and expression of cyanobacterial ATP synthase genes. *Photosynthesis Research*, *18*(1–2), 223–244. https://doi.org/10.1007/BF00042986/METRICS

Da Silva, W. M., Oliveira, L. C., Soares, S. C., Sousa, C. S., Tavares, G. C., Resende, C. P., Pereira, F. L., Ghosh, P., Figueiredo, H., & Azevedo, V. (2019). Quantitative proteomic analysis of the response of probiotic putative *Lactococcus lactis* NCDO 2118 strain to different oxygen availability under temperature variation. *Frontiers in Microbiology*, *10*(APR), 759. https://doi.org/10.3389/FMICB.2019.00759

D'Abrosca, G., Paladino, A., Cuoco, E., Marasco, R., Pacifico, S., Piccolella, S., Vastano, V., Sacco, M., Isernia, C., Muscariello, L., & Malgieri, G. (2018). Structural Characterization of the *Lactobacillus plantarum* FlmC Protein Involved in Biofilm Formation. *Molecules 2018, Vol. 23, Page 2252*, *23*(9), 2252. https://doi.org/10.3390/MOLECULES23092252

Distler, M. G., & Palmer, A. A. (2012). Role of glyoxalase 1 (Glo1) and methylglyoxal (MG) in behavior: Recent advances and mechanistic insights. *Frontiers in Genetics*, *3*(NOV), 250. https://doi.org/10.3389/FGENE.2012.00250

Harvey, K. L., Jarocki, V. M., Charles, I. G., & Djordjevic, S. P. (2019). The diverse functional roles of elongation factor tu (Ef-tu) in microbial pathogenesis. *Frontiers in Microbiology*, *10*(OCT), 2351. https://doi.org/10.3389/FMICB.2019.02351

He, J., Miyazaki, H., Anaya, C., Yu, F., Yeudall, W. A., & Lewis, J. P. (2006). Role of *Porphyromonas gingivalis* FeoB2 in metal uptake and oxidative stress protection. *Infection and Immunity*, *74*(7), 4214–4223. https://doi.org/10.1128/IAI.00014-06

He, M., & Shi, B. (2017). Gut microbiota as a potential target of metabolic syndrome: the role of probiotics and prebiotics. *Cell & Bioscience*, *7*(1). https://doi.org/10.1186/S13578-017-0183-1

Herbel, S. R., Vahjen, W., Wieler, L. H., & Guenther, S. (2013). Timely approaches to identify probiotic species of the genus *Lactobacillus*. *Gut Pathogens*, *5*(1). https://doi.org/10.1186/1757-4749-5-27

Hillas, P. J., Soto Del Alba, F., Oyarzabal, J., Wilks, A., & Ortiz De Montellano, P. R. (2000). The AhpC and AhpD Antioxidant Defense System of *Mycobacterium tuberculosis*. *Journal of Biological Chemistry*, *275*(25), 18801–18809. https://doi.org/10.1074/JBC.M001001200

Liu, X., Omar, M., Abrahante, J. E., Nagaraja, K. V., & Vidovic, S. (2020). Insights into the Oxidative Stress Response of *Salmonella enterica* serovar Enteritidis Revealed by the Next Generation Sequencing Approach. *Antioxidants 2020, Vol. 9, Page 849*, *9*(9), 849. https://doi.org/10.3390/ANTIOX9090849

Petrov, L., Tzvetanova, E., Pavlova, A., Alexandrova, A., Zamfirova, R., Kirkova, M., & Todorov, S. (2010). In-vivo effects of nociceptin and its structural analogue [Orn9] nociceptin on the antioxidant status of rat blood and liver after carrageenan-induced paw inflammation. *Central European Journal of Medicine*, *5*(1), 123–131. https://doi.org/10.2478/S11536-009-0117-3

Queraltó, C., Álvarez, R., Ortega, C., Díaz-Yáñez, F., Paredes-Sabja, D., & Gil, F. (2023). Role and Regulation of Clp Proteases: A Target against Gram-Positive Bacteria. *Bacteria 2023, Vol. 2, Pages 21-36*, *2*(1), 21–36. https://doi.org/10.3390/BACTERIA2010002

Saini, A., Mapolelo, D. T., Chahal, H. K., Johnson, M. K., & Outten, F. W. (2010). SufD and SufC ATPase activity are required for iron acquisition during in vivo Fe-S cluster formation on SufB. *Biochemistry*, *49*(43), 9402–9412. https://doi.org/10.1021/BI1011546

Smaldone, G. T., & Helmann, J. D. (2007). CsoR regulates the copper efflux operon copZA in Bacillus subtilis. *Microbiology*, *153*(12), 4123–4128 https://doi.org/10.1099/MIC.0.2007/011742-0

Spaans, S. K., Weusthuis, R. A., van der Oost, J., & Kengen, S. W. M. (2015). NADPH-generating systems in bacteria and archaea. *Frontiers in Microbiology*, *6*(JUL), 742. https://doi.org/10.3389/FMICB.2015.00742

Stergiou, O. S., Tegopoulos, K., Kiousi, D. E., Tsifintaris, M., Papageorgiou, A. C., Tassou, C. C., Chorianopoulos, N., Kolovos, P., & Galanis, A. (2021). Whole-Genome Sequencing,

Phylogenetic and Genomic Analysis of *Lactiplantibacillus pentosus* L33, a Potential Probiotic Strain Isolated from Fermented Sausages. *Frontiers in Microbiology*, *12*, 3295. https://doi.org/10.3389/FMICB.2021.746659

Susmitha, A., Bajaj, H., & Madhavan Nampoothiri, K. (2021). The divergent roles of sortase in the biology of Gram-positive bacteria. *The Cell Surface*, *7*, 100055. https://doi.org/10.1016/J.TCSW.2021.100055

Teramoto, H., Inui, M., & Yukawa, H. (2013). OxyR acts as a transcriptional repressor of hydrogen peroxide-inducible antioxidant genes in *Corynebacterium glutamicum* R. *The FEBS Journal*, *280*(14), 3298–3312. https://doi.org/10.1111/FEBS.12312

Torrents, E. (2014). Ribonucleotide reductases: Essential enzymes for bacterial life. *Frontiers in Cellular and Infection Microbiology*, *4*(APR), 52. https://doi.org/10.3389/FCIMB.2014.00052

Tsuji, S., Tsuji, N., Sadeghpour Heravi, F., & Hu, H. (2023). Bifidobacterium: Host–Microbiome Interaction and Mechanism of Action in Preventing Common Gut-Microbiota-Associated Complications in Preterm Infants: A Narrative Review. *Nutrients 2023, Vol. 15, Page 709*, *15*(3), 709. https://doi.org/10.3390/NU15030709
